# Genomic epidemiology and antimicrobial resistance of non-typhoidal *Salmonella* in children in Hainan Province, China

**DOI:** 10.1128/spectrum.01502-26

**Published:** 2026-09-23

**Authors:** Huaiyu Jia, Tao Chen, Xiaozhuang Zhang, Guozhen Tian, Min Wang

**Affiliations:** 1 Hainan Women and Children's Medical Center 590865, Haikou, China; 2 Department of Clinical Laboratory, Zhejiang Cancer Hospital 89680, Hangzhou, Zhejiang, China; 3 State Key Laboratory for Diagnosis and Treatment of Infectious Diseases, National Clinical Research Center for Infectious Diseases, China-Singapore Belt and Road Joint Laboratory on Infection Research and Drug Development, National Medical Center for Infectious Diseases, Collaborative Innovation Center for Diagnosis and Treatment of Infectious Diseases, The First Affiliated Hospital, Zhejiang University School of Medicine 26441, Hangzhou, China; Health Canada, Ottawa, Canada; Zhejiang University, Hangzhou, China

**Keywords:** Non-typhoidal *Salmonella* (NTS), Children, Serotype, Antimicrobial Resistance, Molecular Epidemiology

## Abstract

Non-typhoidal *Salmonella* (NTS) is a major foodborne pathogen causing significant morbidity and mortality worldwide, particularly affecting children under 5 years of age. Despite its public health importance, systematic studies on NTS infection in children in Hainan Province, China, are limited. We performed a comprehensive retrospective analysis of 77 NTS isolates collected from children at the Hainan Women and Children’s Medical Center between April 2023 and May 2024. The study integrated antimicrobial susceptibility testing, whole-genome sequencing, and phylogenetic reconstruction to elucidate the molecular epidemiology and resistance of this pathogen. The invasive infection rate of NTS was 10.4% (8/77; 95% CI: 4.6%–19.4%). Children under 3 years were disproportionately affected (66/77, 85.7%), and infections peaked in summer and autumn. Sixteen serotypes and 16 sequence types (STs) were identified. The predominant serotypes were monophasic 1,4,[5],12:i:- (31/77, 40.3%), Typhimurium (12/77, 15.6%), and Stanley (9/77, 11.7%). Serotype was strongly associated with ST, with monophasic 1,4,[5],12:i:- (ST34) (40.3%) and Typhimurium (ST19) (15.6%) being the most prevalent. The ST34 lineage demonstrated extensive multidrug resistance (23/31, 74.2%; 95% CI: 55.4%—88.1%) and accounted for the majority of resistance to ampicillin (29/31, 93.5%; 95% CI: 78.6%—99.2%) and ampicillin-sulbactam (24/31, 77.4%; 95% CI: 58.9%—90.4%). High-risk plasmids were identified, including IncHI2A-type vectors carrying the *bla*_NDM-5_ carbapenemase gene and an IncFIA/HI1 plasmid harboring the *tet*_(X4)_ tigecycline resistance gene. Phylogeographic analysis indicated that the Hainan ST34 strains belong to a China-specific clade undergoing recent clonal expansion although evidence of international transmission was also detected. These findings reveal a distinct epidemiological pattern in Hainan, marked by the predominance of multidrug-resistant ST34 and an elevated invasive infection rate in young children. Targeted surveillance and “One Health” interventions in tropical regions are urgently needed to limit the spread of high-risk NTS clones.

**IMPORTANCE:**

This study addresses the research gap of pediatric non-typhoidal Salmonella (NTS) in tropical Hainan via comprehensive analysis of 77 clinical isolates. It characterizes the local molecular epidemiology, revealing regional epidemic features and high susceptibility in young children. The multidrug-resistant monophasic ST34 clone is identified as the predominant strain. Notably, high-risk resistance plasmids carrying *bla*_NDM-5_ and *tet*_(X4)_ are detected, indicating hidden horizontal transmission risks of critical antibiotic resistance. Global phylogeny verifies the China-specific expansion of local ST34 strains with cross-regional and zoonotic potential. These findings underscore the severe threat of MDR NTS in tropical island areas, providing key epidemiological and genomic evidence for targeted surveillance, prudent antibiotic administration and One Health strategies, and offering vital references for NTS control in tropical and subtropical regions.

## INTRODUCTION

Non-typhoidal *Salmonella* (NTS) is a major foodborne pathogen transmitted through the fecal-oral route, causing gastrointestinal symptoms ranging from self-limiting diarrhea to severe invasive infections. According to the World Health Organization, NTS causes approximately 93.8 million foodborne illnesses and 155,000 deaths annually worldwide (1), with the highest burden in Southeast Asia and the African region (2, 3). Children under 5 years are disproportionately affected, particularly in low- and middle-income countries, where NTS can cause life-threatening invasive infections including bacteremia, meningitis, and osteoarticular infections (3, 4). Notably, the emergence and global spread of multidrug-resistant NTS, especially strains resistant to third-generation cephalosporins, fluoroquinolones, and even carbapenems, has severely limited treatment options and increased mortality risk in pediatric populations (5–7).

The China National Center for Food Safety Risk Assessment (CFSA) estimated the incidence of NTS-associated foodborne gastroenteritis in China at 1,295.59 per 100,000 population, markedly exceeding the burden in developed countries such as the United States (8, 9). Despite this substantial disease burden, NTS infection remains an underrecognized public health priority. Hainan Province, China’s only tropical island, has a year-round warm climate that may facilitate pathogen survival and transmission, distinguishing its epidemic patterns from inland regions. However, systematic studies on NTS molecular epidemiology and resistance mechanisms in this region are lacking.

This study presents a comprehensive genomic analysis of NTS isolates from children in Hainan Province, integrating epidemiological data, antimicrobial susceptibility profiles, resistance gene characterization, and phylogenetic analysis. Our findings yield important insights into the local transmission dynamics of dominant clones such as ST34, as well as the emergence of carbapenem- and tigecycline-resistant strains. These insights can inform targeted prevention strategies and improve clinical management of NTS infections in tropical regions.

## MATERIALS AND METHODS

### Collection of isolates and clinical information

This retrospective study was conducted from April 2023 to May 2024 at Hainan Women and Children’s Medical Center, the sole provincial tertiary hospital guiding maternal and child healthcare across Hainan. The study population comprised pediatric patients with confirmed NTS infection. Clinical specimens were processed using standard microbiological methods. Stool samples were inoculated onto Columbia blood agar, xylose lysine deoxycholate agar, and thiosulfate citrate bile salts sucrose agar (all from Guangzhou Dijing Microbial Technology Co., Ltd.) and incubated at 35°C in 5% CO_2_ for 18 to 24 h. Blood samples were cultured using the VITEK-Compact system (bioMérieux, Marcy-l'Étoile, France). Patient information, including sex, age, infection site, specimen collection date, and attending department, was retrieved from the electronic medical record system with personal identifiers removed. This study was approved by the Ethics Committee of Hainan Women and Children’s Medical Center (approval number: HNWCMC-2026-110).

### Antimicrobial susceptibility testing

Species identification was performed using the VITEK-Compact 2 automated microbial identification system (bioMérieux, Marcy-l'Étoile, France). Antimicrobial susceptibility was assessed using the VITEK Gram-negative susceptibility card (GN13) for 11 agents: ampicillin (AMP), ampicillin-sulbactam (SAM), aztreonam (ATM), ceftazidime (CAZ), ceftriaxone (CRO), ciprofloxacin (CIP), ertapenem (ETP), imipenem (IMP), levofloxacin (LVX), piperacillin-tazobactam (TZP), and trimethoprim-sulfamethoxazole (SXT). CIP susceptibility was confirmed by disk diffusion (Kirby-Bauer method) using 5 μg disks (Wenzhou Kangtai Biological Products Co., Ltd., China). Results were interpreted according to the Clinical and Laboratory Standards Institute (CLSI) M100 36th edition (2026) and the European Committee on Antimicrobial Susceptibility Testing (EUCAST) 2026 v16.0 breakpoints (10, 11). *Escherichia coli* (*E. coli*) ATCC 25922 was used for quality control. Multidrug resistance (MDR) was defined as resistance to three or more antimicrobial agents (12).

### Whole-genome sequencing, MLST, and serotyping

All 77 isolates were sequenced using an Illumina HiSeq 2500 platform (2 × 125 bp paired-end reads). *De novo* assembly was performed with SPAdes v3.10.0. Multilocus sequence typing (MLST) was assigned using mlst (https://github.com/tseemann/mlst). MOB-suite v1.4.9 (https://github.com/phac-nml/mob-suite) was used to reconstruct and characterize the plasmids. For isolates S6, S20, S55, and S76 (carrying *bla*_NDM-5_, *tet*_(X4)_, or two copies of *bla*_TEM-1_), additional long-read sequencing was conducted on the Oxford Nanopore MinION platform. Hybrid assemblies were generated with Unicycler v0.4.8 (13) and annotated using Prokka v1.13 (14). Assembly quality metrics are reported in Table S1. Serovar prediction was performed using SeqSero2 v1.2.1 (15) and SISTR v1.1.1 (16).

### Phylogenetic analysis

A pangenome analysis and core gene alignment for all 77 Salmonella enterica isolates were generated using Roary v3.11.2 with default parameters (17). A maximum-likelihood phylogeny was inferred with RAxML v8.2.12 under the GTR + GAMMA model with 100 rapid bootstrap replicates. A total of 687 publicly available *Salmonella enterica* ST34 genomes were sourced from PATRIC. These genomes were subjected to quality control and screened for metadata completeness, resulting in a final data set of 660 isolates for comparative analysis (details in Table S2). Core-genome SNPs for ST34 isolates were identified using Snippy v4.6.0, with the complete genome of ST34 strain R17.5474 (GenBank: GCA_024266445.1) as reference. Recombination regions were removed using Gubbins v2.4.1, and a recombination-free core-SNP alignment was used to construct a maximum-likelihood tree in IQ-TREE v2.3.6 with GTR + GAMMA model (18).

### Antimicrobial resistance and plasmid profiling

Acquired antimicrobial resistance genes and chromosomal resistance mutations were identified using AMRFinderPlus v3.10.23 with default settings (19). Plasmid replicon types were determined against the PlasmidFinder database via ABRicate v1.0.1.

### Phylodynamic analysis of ST34

To evaluate temporal signal, root-to-tip regression was performed in TempEst v1.5.3.

A total of 139 genomes were selected using a stratified sampling strategy to ensure the representation of Hainan isolates and a balanced temporal distribution across the study period, while maintaining computational feasibility for Bayesian analysis. A recombination-free core-genome alignment (generated with Snippy and Gubbins) was analyzed in BEAST v1.10.4 under a Bayesian Markov Chain Monte Carlo (MCMC) framework (20), using sample collection dates for tip calibration. The most appropriate demographic model for this study was defined as the GTR nucleotide substitution model with four gamma categories, combined with an uncorrelated relaxed clock model. We employed a Bayesian Skyline coalescent tree prior to allow for variable population size through time. MCMC convergence was assessed by effective sample size (ESS > 200) in Tracer v1.7.1. The maximum clade credibility tree was summarized with TreeAnnotator v1.10.4 and visualized in FigTree v1.4.4.

### Statistical analysis

Statistical analyses were conducted using Microsoft Excel 2019 and GraphPad Prism 10. Categorical variables were compared with Fisher’s exact test or the chi-square test, and pairwise comparisons among multiple groups were adjusted by the Bonferroni method. The concordance between serotype and ST was quantified using Cramér’s V. The exact binomial test was used to compare the observed male proportion against the expected 50%; visit type and sample type were assessed similarly. Goodness-of-fit *χ*² tests were used to compare ward and age group distributions against uniform distributions. Clopper-Pearson exact 95% confidence intervals (CI) were calculated for all proportions. A two-sided *P* < 0.05 was considered statistically significant.

## RESULTS

### Demographic features of NTS infection in children

From April 2023 to May 2024, 77 non-duplicate NTS strains were collected from outpatient and hospitalized children. Of these, 69 (89.6%) were isolated from fecal samples and 8 (10.4%) from blood samples, yielding an invasive infection rate of 10.4%. Most strains were recovered from the gastroenterology ward (24/77, 31.2%), followed by the outpatient department (15/77, 19.5%), infection ward (8/77, 10.4%), and hematology ward (8/77, 10.4%). Among the infected children, males accounted for 43 cases (55.8%) and females for 34 cases (44.2%). Isolates were unevenly distributed across age groups, with 85.7% (66/77) recovered from children under 3 years of age and limited representation in the 4–6 years group (3.9%, 3/77) and the 7–18 years group (10.4%, 8/77). The epidemic season was predominantly summer and autumn (Table 1).

**TABLE 1:** Demographic features of children with NTS infection, Haikou, China

Clinical parameters	Total (*n*)	Ratio (%)	*P*
Type of visit			
Hospitalization	62	80.5	<0.001
Outpatient service	15	19.5	
Sample type			
Fecal	69	89.6	<0.001
Blood	8	10.4	
Ward distribution^*a*^			
Gastroenterology	24	31.2	0.0028
Outpatient department	15	19.5	
Infection ward	8	10.4	
Hematology ward	8	10.4	
Neurology	6	7.8	
Others	16	20.8	
Sex			
Males	43	55.8	0.3082
Females	34	44.2	
Age group^*b*^			
0–3 years	66	85.7	<0.001
4–6 years	3	3.9	
7–18 years	8	10.4	
Season^*c*^		% (a/A)	
Spring	7	3.7 (7/189)	0.0181
Summer	30	7.8 (30/386)	
Autumn	29	10.4 (29/278)	
Winter	11	4.8 (11/229)	

^*a*^ The ward distribution differed significantly from uniformity (*χ*² = 18.14, *P* = 0.0028), with gastroenterology being the only department exceeding the expected proportion [standardized residual (SR) = 3.12)].

^*b*^ The age distribution departed significantly from uniformity (*χ*² = 95.56, *P* < 0.001), with cases concentrated in the 0–3 years group (SR = 7.96) and underrepresented in the 4–6 years and 7–18 years groups (SR = −4.47 and −3.49, respectively).

^*c*^ The ratio in different seasons represents the detection rate of NTS in fecal culture specimens; a indicates the number of NTS-positive fecal isolates, and A indicates the total number of fecal culture specimens. Seasonal detection rates differed significantly (*χ*² = 10.06, *P* = 0.0181); only the spring-autumn comparison remained significant after Bonferroni correction.

### Antimicrobial susceptibility of NTS

Antimicrobial susceptibility testing revealed high resistance rates to commonly used antibiotics (Table 2). The highest resistance rates were observed for AMP (54/77, 70.1%; 95% CI: 58.6%–80.0%) and SAM (47/77, 61.0%; 95% CI: 49.2%–72.0%), followed by SXT (38/77, 49.4%; 95% CI: 37.8%–61.0%). Resistance to third-generation cephalosporins was moderate: CRO 19/77 (24.7%; 95% CI: 15.6%–35.8%) and CAZ 16/77 (20.8%; 95% CI: 12.4%–31.5%). Low-level carbapenem resistance was detected, with four isolates resistant to ETP (5.2%; 95% CI: 1.4%–12.8%) and three to IPM (3.9%; 95% CI: 0.8%–11.0%). Fluoroquinolone resistance varied by agent: CIP showed 15/77 resistance (19.5%; 95% CI: 11.3%–30.1%) with 24/77 (31.2%; 95% CI: 21.1%–42.7%) intermediate susceptibility, whereas LVX demonstrated only 1/77 (1.3%; 95% CI: 0.0%–7.0%) resistance but 27/77 (35.1%; 95% CI: 24.5%–46.8%) intermediate susceptibility. TZP and ATM maintained relatively high susceptibility at 90.9% (70/77; 95% CI: 82.2%–96.3%) and 79.2% (61/77; 95% CI: 68.5%–87.6%), respectively.

**TABLE 2:** Antimicrobial susceptibility pattern of NTS strains^*a*^

Antimicrobials	*R* (%)	*I* (%)	*S* (%)	*R* (%) 95% CI
AMP	70.1	0	29.9	58.6–80.0
SAM	61.0	6.5	32.5	49.2–72.0
SXT	49.4	0	50.6	37.8–61.0
CRO	24.7	0	75.3	15.6–35.8
CAZ	20.8	0	79.2	12.4–31.5
ATM	20.8	0	79.2	12.4–31.5
CIP	19.5	31.2	49.3	11.3–30.1
TZP	9.1	0	90.9	3.7–17.8
ETP	5.2	0	94.8	1.4–12.8
IPM	3.9	0	96.1	0.8–11.0
LVX	1.3	35.1	63.6	0.0–7.0

^*a*^ R, Resistant; I, intermediate resistance; S, susceptible; AMP, ampicillin; SAM, ampicillin-sulbactam; CRO, ceftriaxone; CAZ, ceftazidime; ETP, ertapenem; IPM, imipenem; CIP, ciprofloxacin; LVX, levofloxacin; SXT, trimethoprim-sulphamethoxazole; TZP, piperacillin-tazobactam; ATM, aztreonam.

### Serotype distribution of NTS

**Fig 1:**
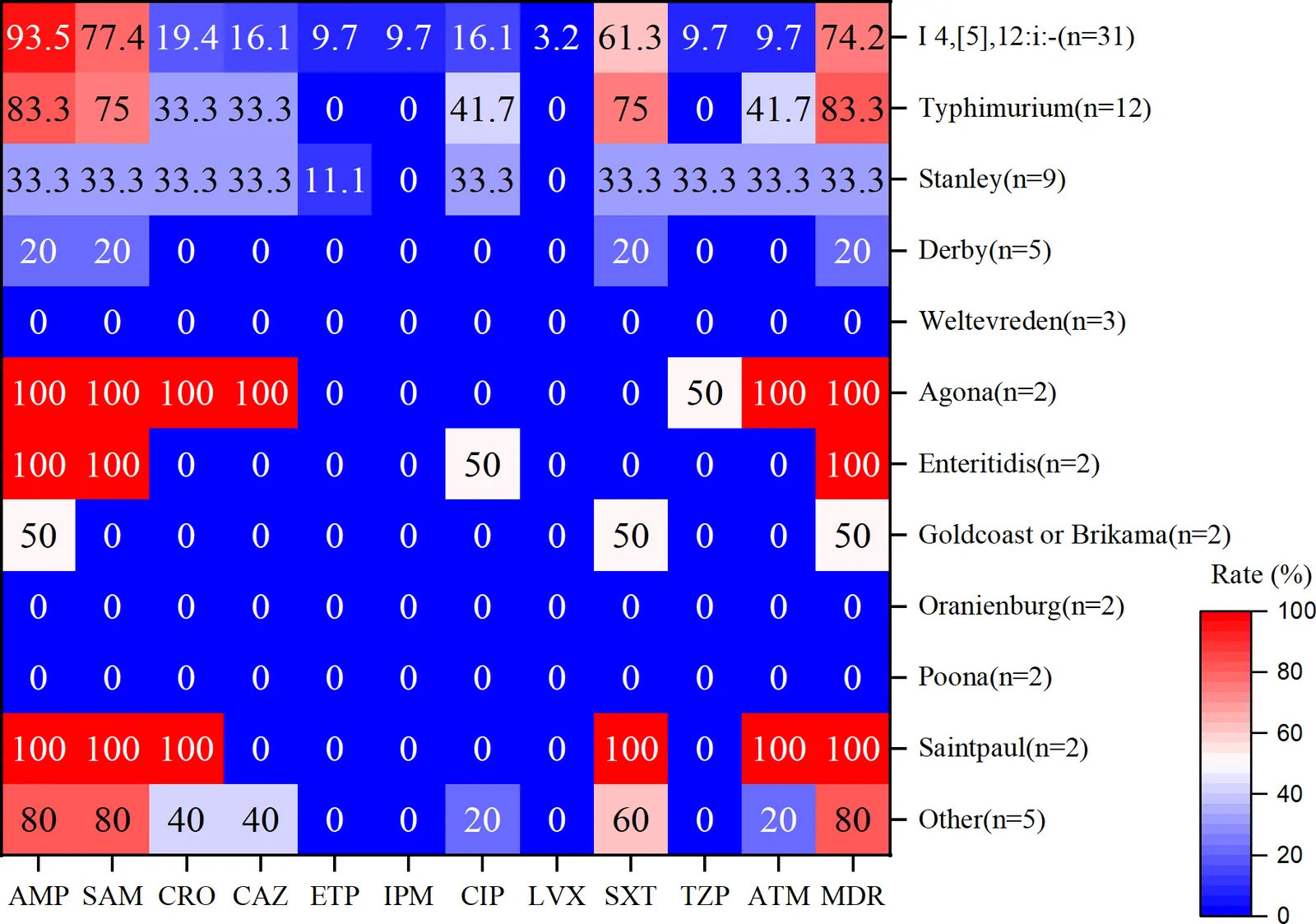
Antimicrobial resistance rates by serotype in NTS. AMP, ampicillin; SAM, ampicillin-sulbactam; ATM, aztreonam; CAZ, ceftazidime; CRO, ceftriaxone; CIP, ciprofloxacin; ETP, ertapenem; IPM, imipenem; LVX, levofloxacin; TZP, piperacillin-tazobactam; SXT, trimethoprim-sulphamethoxazole; MDR, multidrug resistance. Other serotypes included Rissen, Albany or Duesseldorf, Litchfield, Javiana, and Liverpool. Statistical comparison of resistance rates was limited to monophasic 1,4,[5],12:i:- (ST34) and Typhimurium (ST19), the only serotypes with >10 isolates. Resistance proportions for remaining serotypes (*n* < 10) are presented with exact 95% CI and should be interpreted with caution. Detailed results are provided in Table S3.

A total of 16 distinct serotypes were identified among the 77 NTS isolates. The predominant serotype was monophasic 1,4,[5],12:i:- (*n* = 31, 40.3%), followed by Typhimurium (*n* = 12, 15.6%), Stanley (*n* = 9, 11.7%), Derby (*n* = 5, 6.5%), and Weltevreden (*n* = 3, 3.9%). Rare serotypes including Agona, Enteritidis, Goldcoast or Brikama, Oranienburg, Poona, and Saintpaul each accounted for 2/77 (2.6%), while five other serotypes were grouped as “Other” (*n* = 5, 6.5%) (Fig. 1).

The predominant serotype monophasic 1,4,[5],12:i:- exhibited high resistance rates to AMP (29/31, 93.5%; 95% CI: 78.6%–99.2%), SAM (24/31, 77.4%; 95% CI: 58.9%–90.4%), and SXT (19/31, 61.3%; 95% CI: 42.2%–78.2%), with moderate resistance to CRO (6/31, 19.4%; 95% CI: 7.5%–37.5%) and CAZ (5/31, 16.1%; 95% CI: 5.5%–33.7%). Typhimurium demonstrated a similar resistance pattern, with high rates of AMP (10/12, 83.3%; 95% CI: 51.6%–97.9%), SAM (9/12, 75.0%; 95% CI: 42.8%–94.5%), and SXT (9/12, 75.0%; 95% CI: 42.8%–94.5%) resistance. Resistance proportions for serotypes with *n* < 10 are reported with exact 95% CI and should be interpreted with caution owing to small sample sizes (Fig. 1 and Table S3).

### MLST of NTS isolates

**Fig 2:**
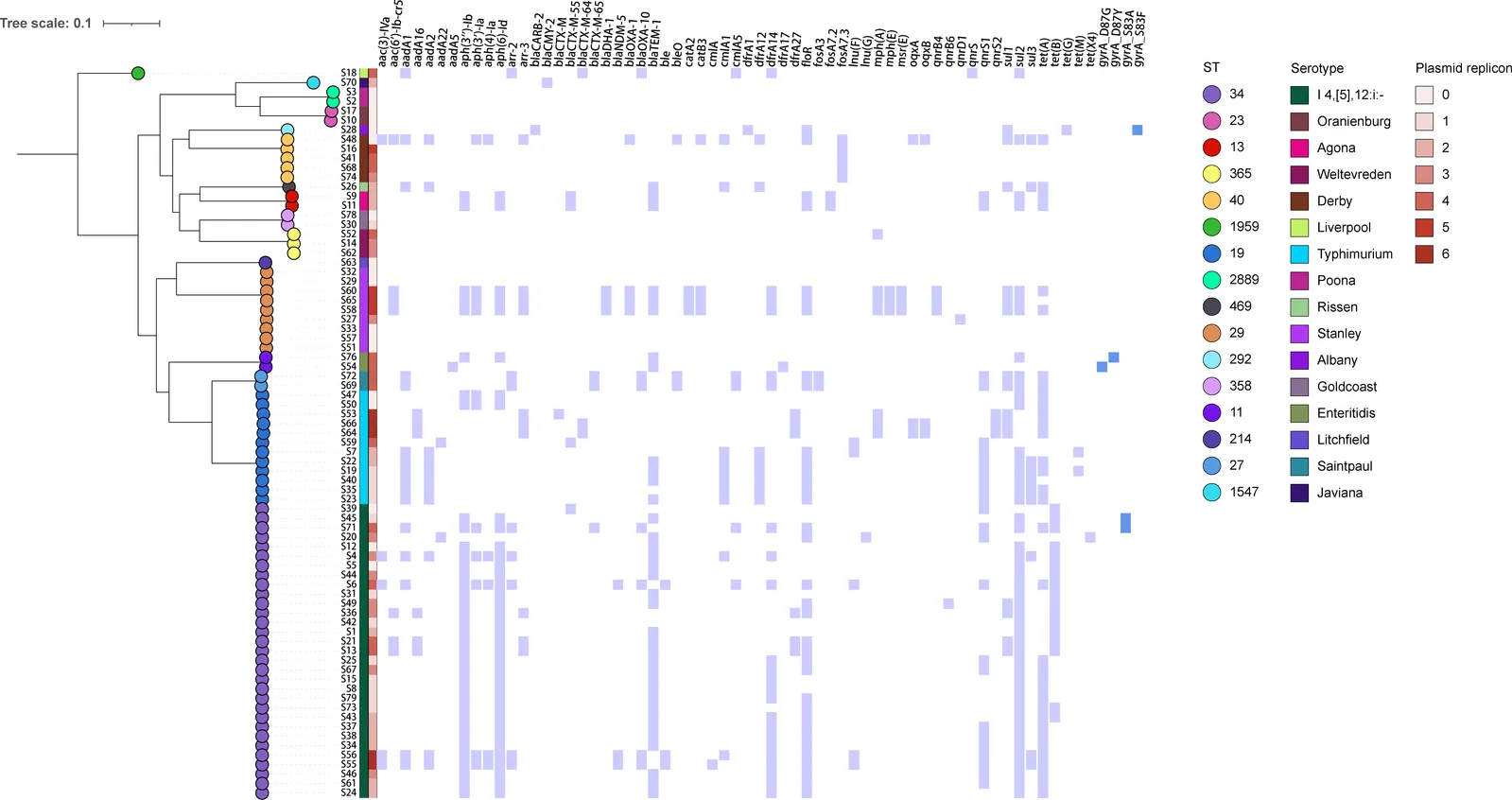
Maximum-likelihood phylogenetic tree of NTS (*n* = 77). Metadata displayed in the accompanying tracks include ST, serotype, plasmid replicon number, and the presence/absence of antimicrobial resistance genes.

A high diversity of sequence types (STs) was observed among the 77 NTS isolates, with 16 different STs identified. Five STs each represented more than 3% of the population: ST34 (*n* = 31, 40.3%), ST19 (*n* = 12, 15.6%), ST29 (*n* = 9, 11.7%), ST40 (*n* = 5, 6.5%), and ST365 (*n* = 3, 3.9%), collectively accounting for 77.9% of isolates. Serotype and ST exhibited a perfect one-to-one correspondence (Cramér’s *V* = 1.000); monophasic 1,4,[5],12:i:- (ST34) and Typhimurium (ST19) were the most prevalent serotypes (Table S4). Phylogenetic analysis revealed a diverse population composed of distinct lineages, with the most prominent clones linked to the ST34 and ST19 clade (Fig. 2).

### Antimicrobial resistance mechanisms and virulence genes

Resistance profiles varied among STs and within identical STs. Strains harboring a greater number of resistance genes tended to carry more plasmid replicons, suggesting that a substantial proportion of these resistance determinants are likely plasmid-borne (Fig. 2). Notably, three ST34 strains (S55, S56, and S6) harbored *bla*_NDM-5_ carbapenemase genes on IncHI2A plasmids. For strains S55 and S6, hybrid assembly of short- and long-read sequencing data revealed that the *bla*_NDM-5_ genes were located on distinct plasmids, designated pS6-NDM-5 (247,169 bp) and pS55-NDM-5 (232,164 bp), respectively. The pS6-NDM-5 plasmid carried multiple resistance genes including *aac (3)-IVa*, *qnrS1*, and *bla*_OXA-10_, while pS55-NDM-5 carried *arr-2*, *cmlA*, and *dfrA14*. For strain S56, plasmid reconstruction using Mob-suite predicted that *bla*_NDM-5_ was similarly located on an IncHI2A plasmid. Comparative analysis showed that the plasmids from S55 and S6 shared an identical backbone with high similarity (>90% coverage, 100% identity) to p3M-23 (China) and pZ1323SSL0063-1 (South Korea) (Fig. S1A). However, the latter two lacked the *bla*_NDM-5_-carrying region, which is flanked by mobile genetic elements (*IS26-IS3000-IS5-bla*_NDM-5_-*IS26*), suggesting acquisition of *bla*_NDM-5_ via insertion into a conserved backbone (Fig. S1B). In addition, ST34 strain S20 carried *tet*_(X4)_ on a 186,003-bp IncFIA(HI1)/IncHI1A/IncHI1B(R27) plasmid harboring *floR*, *qnrS1*, and *bla*_TEM-1_, with 99.9% identity to pLJP3-1 (*E. coli*, China) and pTQ28-*tet*_(X4)_ (*Morganella morganii*, China), indicating potential inter-species spread (Fig. S1C). Additionally, ST11 NTS Enteritidis strain S76 harbored two copies of *bla*_TEM-1_ on distinct plasmids (64,327-bp IncFIB(S)/IncFII(S) and 24,484-bp IncX1), with the IncX1 plasmid additionally carrying *aph (6)-Id*, *aph(3")-Ib*, and *sul2*. Both *bla*_TEM-1_ copies were embedded in Tn2 transposons, facilitating horizontal transmission (Fig. S1D).

**Fig 3:**
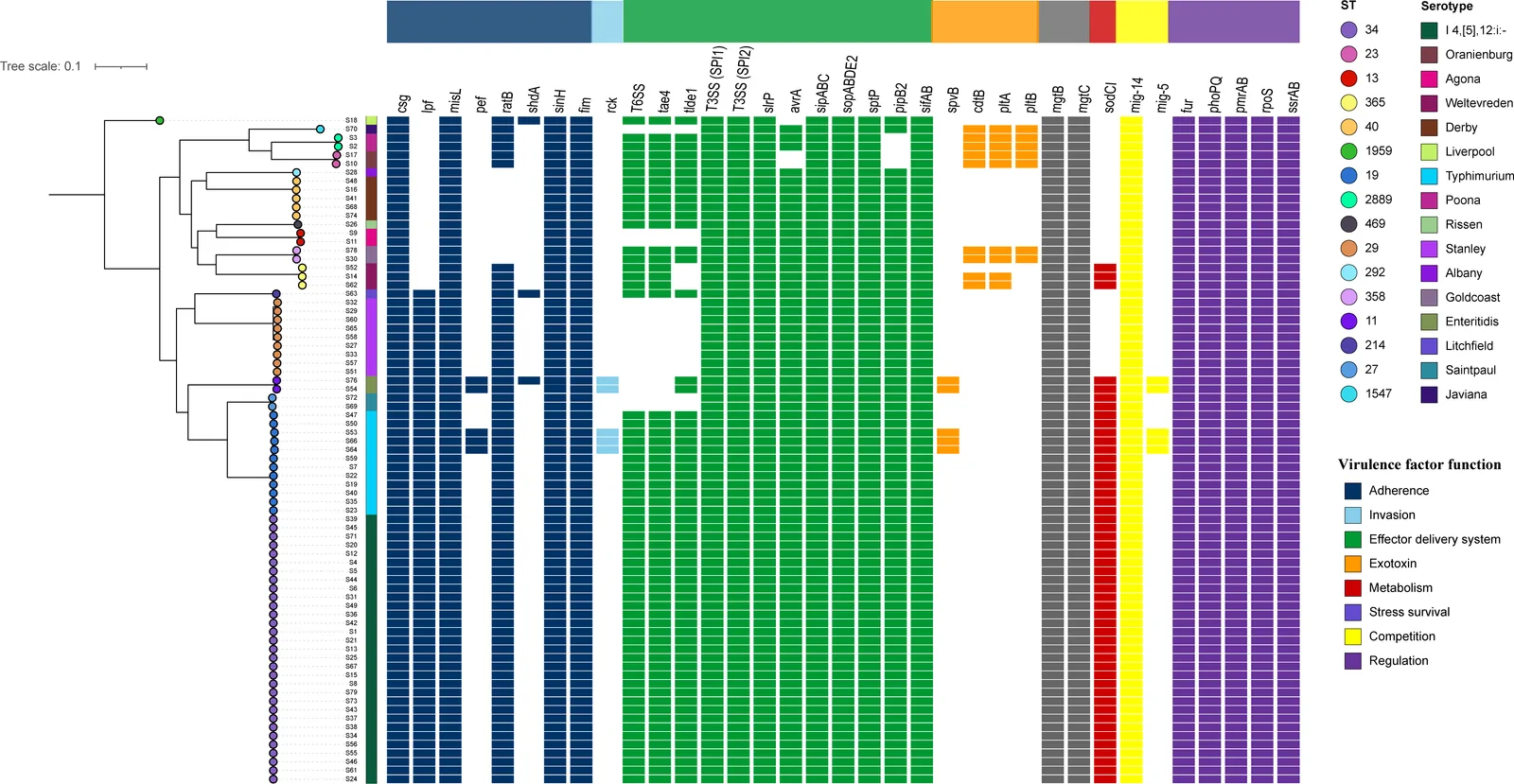
Virulence gene profiles of the NTS isolates (*n* = 77). Each row represents an isolate, and columns represent specific virulence genes clustered by functional categories (color-coded).

Virulence gene distribution was serotype-specific (Fig. 3). Two NTS Enteritidis (S54 and S76) and three NTS Typhimurium strains (S53, S64, and S66) harbored *pef*, *rck*, *spvB*, and *mig-5*. Toxin genes *cdtB*, *pltA*, and *pltB* were present in all NTS Javiana (S70), Poona (S2 and S3), Oranienburg (S10 and S17), and Goldcoast (S30 and S78) strains, whereas *shdA* was restricted to NTS Liverpool (S18), Litchfield (S63), and Enteritidis (S76). Strains of Javiana, Agona, Stanley, Enteritidis, and Saintpaul entirely lacked the Type VI secretion system (T6SS). The complete virulence gene profiles for all 77 isolates are listed in Table S5.

### Phylogeography and temporal evolution of ST34

**Fig 4:**
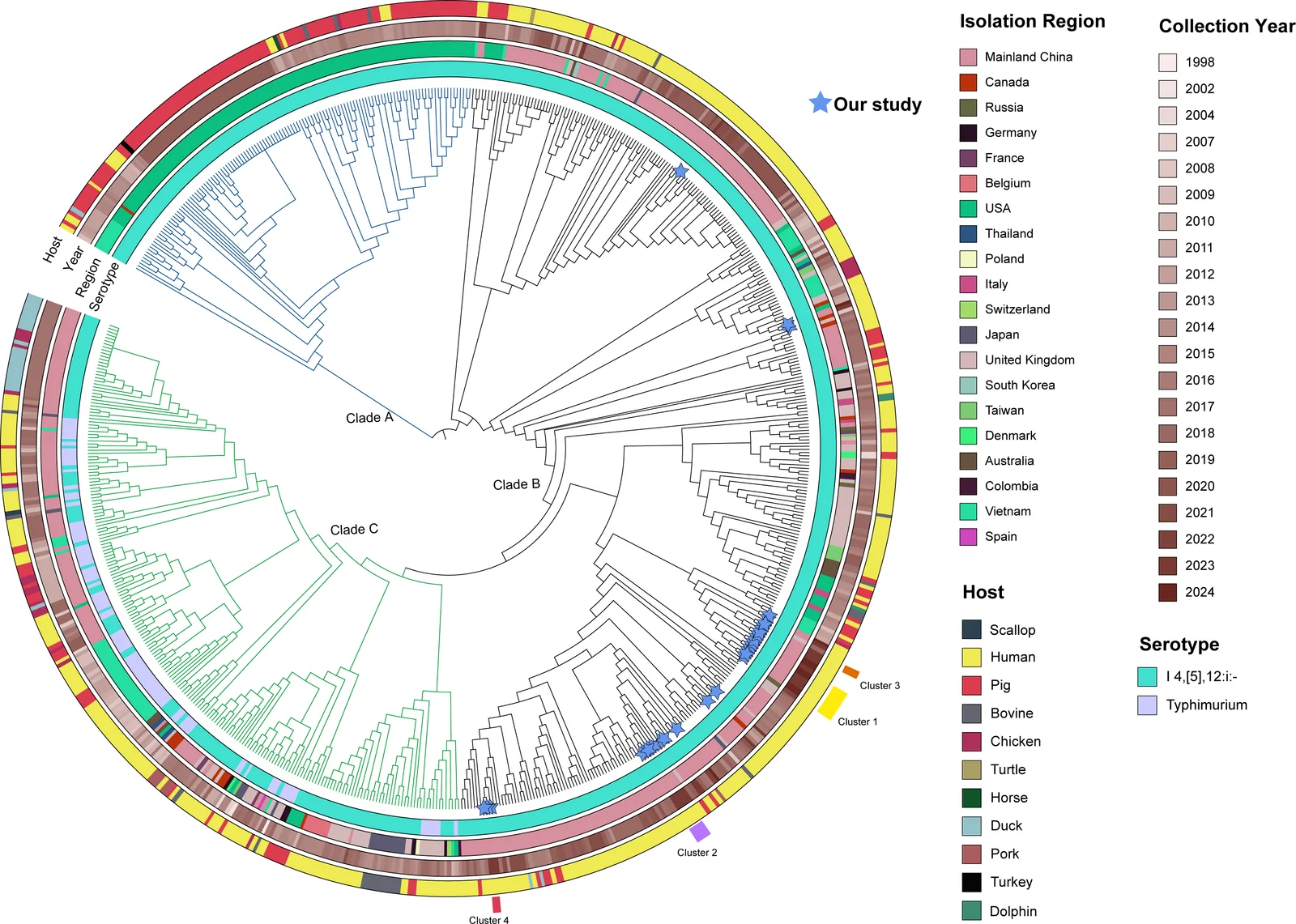
Phylogeographic analysis of a global collection of NTS ST34 isolates (*n* = 691). Metadata, including serotype, geographic region, isolation year, and host source, are visualized as concentric rings from the inner to the outer circle of the tree.

Population dynamics of the predominant ST34 NTS clone were investigated using 660 global NTS genomes from the PATRIC database and 31 isolates from this study (Table S2). These ST34 isolates originated from 20 regions between 1998 and 2023, with Mainland China (314/691, 45.4%), the United States (144/691, 20.8%), the United Kingdom (80/691, 11.6%), and Vietnam (65/691, 9.4%) being the most common sources (Fig. S2). Humans were the primary host (433/691, 62.7%), followed by pigs (172/691, 24.9%). ST34 comprised two serotypes: monophasic 1,4,[5],12:i:- (602/691, 87.1%) and Typhimurium (89/691, 12.9%) and exhibited strong regional epidemiology with consistent geographic clustering of isolates (Fig. 4). Phylogenetic analysis revealed three distinct clades (Clades A–C). Clade A (*n* = 126) comprised isolates from outside China, predominantly from the United States and Vietnam, with most from pigs. Clades B and C primarily comprised human-origin isolates from various regions in China, with Clade C predating Clade B and displaying both serotypes, whereas Clade B comprised only monophasic 1,4,[5],12:i:-, suggesting serotype diversification. Study strains were distributed across Clade B, with several isolates forming four closely related clusters characterized by a pairwise cgSNP distance of <10 SNPs and close temporal clustering, consistent with recent clonal expansion (Fig. 4 and Table S6). Two isolates (S45 and S71) clustered with isolates from the United States, Vietnam, and Canada, indicating potential inter-regional transmission. The intermingling of animal and human isolates highlights the significant risk of zoonotic transmission.

To evaluate temporal signal, root-to-tip regression was performed. The root-to-tip regression analysis showed a temporal signal with a correlation coefficient = 0.6028, *P* < 0.0001 (Fig. S3A). Convergence was confirmed using Tracer, with all effective sample sizes (ESS) exceeding 200 for key parameters, including the clock rate (ESS = 237) and TMRCA (ESS = 264). The most recent common ancestor of ST34 emerged in 1997 (95% HPD interval 1989–2003) in China, with subsequent rapid expansion from 2004 to 2016 followed by a decline since 2017 (Fig. S3B and C).

## DISCUSSION

NTS is one of the most common foodborne gastrointestinal pathogens in China, ranking among the top three bacterial causes of foodborne outbreaks (21). However, epidemiological characteristics of this pathogen remain severely understudied in Hainan, China’s only tropical island province. Here, we characterize the serotypes, antimicrobial resistance, and genomic epidemiology of NTS in children from this region, updating our understanding of local transmission dynamics.

Among the 77 NTS strains investigated, 85.7% were isolated from children aged 0–3 years, indicating that infants and toddlers are disproportionately affected. The summer-autumn peak observed in our study aligns with the national epidemic period (May–September) reported across China (22). The invasive infection rate of 10.4% is among the higher rates reported in recent Chinese pediatric hospital series (23–25). While year-round warm temperatures may facilitate bacterial survival and transmission, direct supporting environmental or food-chain evidence is lacking (26).

The predominant serotypes were monophasic 1,4,[5],12:i:-, Typhimurium, and Stanley, a pattern largely consistent with observations from most other Chinese regions. Notably, Enteritidis accounts for a higher proportion of infections in most provinces, suggesting regional heterogeneity in strain circulation (21, 27–29). Typhimurium and its monophasic variant 1,4,[5],12:i:-, associated with ST19 and ST34, respectively, exhibited MDR to commonly used antibiotics, consistent with global reports (30).

Antimicrobial susceptibility data revealed concerning resistance patterns. The high resistance rates to AMP (70.1%) and SAM (61.0%) render these traditional β-lactams unsuitable for empiric therapy, while moderate resistance to third-generation cephalosporins (CRO 24.7%, CAZ 20.8%) and emerging fluoroquinolone resistance (CIP 19.5% with 31.2% intermediate susceptibility) threaten the efficacy of current WHO-recommended first-line regimens. The detection of carbapenem-resistant isolates, though at low frequency (5.2%), further narrows therapeutic options. Notably, the resistance rate to third-generation cephalosporins in this study was lower than that reported in cities such as Hangzhou and Shenzhen (31, 32), possibly reflecting regional variations in antibiotic consumption or surveillance intensity.

The observed variation in resistance profiles among strains of the same ST is consistent with horizontal gene transfer via plasmid acquisition and loss. The *tet(X4*) gene, encoding a flavin-dependent monooxygenase that inactivates all tetracycline derivatives including tigecycline and eravacycline, was first identified in *E. coli* from food-producing animals in China in 2019 and has since been detected in diverse Enterobacterales species across multiple ecological niches (33, 34). The identification of a *tet(X4*)-harboring mosaic plasmid in our Hainan NTS ST34 isolate, showing high sequence similarity to *tet(X4*)-carrying plasmids previously characterized in *E. coli* and *Morganella* spp. in China, supports inter-species horizontal gene transfer risk. Similarly, the insertion of *bla*_NDM-5_ into a conserved IncHI2A backbone observed in our isolates mirrors the genetic architecture of NDM-5-carrying IncHI2/IncHI2A plasmids recently reported in *Salmonella* serovar Stanley and *E. coli* from food animals in China (35, 36), underscoring the role of these large conjugative plasmids as vehicles for inter-species dissemination of carbapenem resistance. The identification of these resistance determinants on mobile genetic elements with broad host ranges raises concerns about their potential dissemination across bacterial species and geographic boundaries, thereby underscoring the need for integrated surveillance of carbapenem and tigecycline resistance in NTS, given the limited therapeutic options available for invasive infections in children. Although we did not perform experimental conjugation assays in this study, the potential for horizontal dissemination of these plasmids warrants careful consideration in the context of environmental temperature. It has been well established that certain plasmid incompatibility groups, particularly IncHI1-type plasmids, exhibit thermosensitive conjugative transfer with markedly higher frequencies at ambient temperatures (<30°C) compared to 37°C (37). Given that Hainan is characterized by a tropical climate with prolonged periods of elevated ambient temperature, such mechanisms may differentially influence the transfer dynamics of these plasmids across ecological niches. Future studies incorporating temperature-gradient conjugation experiments would be valuable to quantify the transmission potential of the plasmids characterized here under conditions mimicking both environmental and host-associated settings.

More importantly, the occurrence of *bla*_NDM-5_ and *tet*(_X4_) on plasmids within NTS ST34 isolates should be regarded as an early warning signal of the emergence of carbapenem and tigecycline resistance in this high-risk NTS clone in tropical China. NTS ST34 has been recognized as a drug-resistant lineage, and the acquisition of plasmids encoding both NDM-5-mediated carbapenem hydrolysis and Tet(X4)-mediated tigecycline degradation effectively compromises two of the last-resort therapeutic options for salmonellosis (38, 39). This concern is further substantiated by reports from southern China, where various *bla*_NDM-1/5_-carrying plasmids were characterized from ST34 clinical and animal-associated environmental strains (40, 41).

Our phylogeographic analysis places the ST34 Hainan isolates within a global context. Clade A comprised predominantly pig-origin isolates from the United States and Vietnam, suggesting zoonotic transmission at the animal-human interface. In contrast, Clade B, which contained all our study strains, consisted almost exclusively of human-origin isolates from China, indicating sustained local clonal expansion. Recent genomic studies have elucidated evolutionary signatures associated with the pandemic spread of ST34, including sequential gene loss events that may promote clonal fitness and host adaptation. Specifically, the stepwise deletion of prophage regions (such as Gifsy-1 and HP1), combined with IS26-mediated microevolution around the *fljAB* locus, has been shown to generate clonally expanding sublineages with enhanced adaptability to multiple host species (42, 43). Additionally, the acquisition of prophages carrying *sopE* and *perC* genes, which facilitate epithelial cell invasion and nitrate reduction in the gut lumen, along with the integration of the SGI-4 and Tn21 transposon-mediated resistance to heavy metals and antibiotics, supports the persistence of ST34 in intensive livestock farming environments and confers a selective advantage to epidemic lineages (44). Conversely, the intracellular macrophage virulence activator *cspC* is one of the most frequently degraded targets in ST34. As this RNA chaperone is essential for activating the PhoQP virulence regulatory network, its degradation suggests that ST34 is evolving toward attenuated virulence and reduced host invasion (45). Future comparative genomic analyses incorporating long-read sequencing of representative clade B isolates would be valuable to determine whether these Hainan strains carry the same gene loss signatures and prophage dynamics that have been associated with pandemic spread of ST34 in other regions.

The European clone of ST34 *Salmonella* 1,4,[5],12:i:- is estimated to have emerged around 1980 (44). Our calculated TMRCA of 1997 for the Chinese ST34 lineage is slightly later, a finding that mirrors results from a recent large-scale study in Southeast Asia. In that study, temporal phylogenetic inference estimated the MRCA of the regional ST34 population to be 1995 (95% HPD: 1992–1998) (45). Furthermore, our Bayesian Skyline Plot analysis indicated a decline in the effective population size of ST34 after 2017. Notably, China implemented stringent national action plans to contain antimicrobial resistance around 2016–2017, including a ban on the use of colistin as a growth promoter in animal feed. These interventions may have reduced the selective advantage of multidrug-resistant ST34 strains, leading to a contraction in population size. However, continued surveillance is required to disentangle the effects of sampling bias from genuine epidemiological shifts.

In light of these observations, we recommend that surveillance programs for antimicrobial-resistant Salmonella in China and similar tropical regions incorporate molecular screening thresholds for high-risk mobile genetic elements. Specifically, routine genomic surveillance should prioritize the detection of *bla*_NDM_-carrying plasmids and *tet(X4*)-harboring plasmids, particularly when identified in ST34 or other internationally disseminated clones. The establishment of predefined alert thresholds, such as the detection of these plasmids in more than a defined proportion of sentinel surveillance isolates, or their identification in food animal or environmental reservoirs, could facilitate early intervention before widespread clonal expansion occurs. In addition, we strongly advocate for the implementation of integrated human-animal-food surveillance programs in tropical island settings such as Hainan, aligned with the objectives of China’s National Action Plan on Containing Antimicrobial Resistance (2022–2025). Furthermore, given that infants and young children represent the most vulnerable population for foodborne salmonellosis (29), enhanced surveillance of NTS in infant food products and the integration of long-read sequencing into routine monitoring workflows will be essential for tracking their inter-host transmission pathways across the human-animal-food-environment interface in tropical island ecosystems.

This study has several limitations. First, as a single provincial tertiary pediatric referral center receiving severe cases from across Hainan, our 77-isolate cohort may not fully represent the broader pediatric population, and the invasive infection rate (10.4%) may be overestimated owing to upward referral bias. Second, resistance estimates for rare serotypes (*n* = 2–5) are imprecise owing to limited sample sizes. Third, the absence of concurrent food, animal, and environmental isolates prevented direct source attribution and transmission tracing. Finally, due to the high computational demand of Bayesian coalescent frameworks, we subsampled the data set to 139 representative genomes. To mitigate potential sampling bias, we utilized a stratified sampling approach that prioritized the inclusion of Hainan isolates and ensured a balanced temporal distribution of sequences throughout the study period. However, this subsampling strategy may not capture the full extent of the genetic diversity present in the broader population. Consequently, while our estimates for the TMRCA and population dynamics provide valuable insights into the ST34 lineage in this region, they should be interpreted with the understanding that larger-scale genomic surveillance might reveal finer sub-structures or slightly different emergence timelines.

Future work should adopt a One Health approach, including (i) sentinel surveillance linking pediatric hospitals to veterinary and food-safety laboratories in Hainan and Guangdong; (ii) integration of NTS whole-genome sequencing into national AMR surveillance; (iii) targeted monitoring of *bla*_NDM-5_ and *tet*_(X4)_-carrying plasmids in the food chain, particularly poultry and pork production; and (iv) longitudinal tracking of resistance trends and plasmid epidemiology to guide empirical therapy (46, 47).

### Conclusions

This study presents a comprehensive genomic analysis of NTS in children from Hainan Province. Phylogeographic analysis placed Hainan isolates within a globally disseminated ST34 lineage, with evidence of local clonal expansion and potential inter-regional transmission. These findings support the need for enhanced molecular surveillance, antimicrobial stewardship, and integrated One Health strategies to monitor and control the spread of resistant NTS in tropical regions.

## Supplementary Material

Reviewer comments

## Data Availability

All sequencing data generated in this work are available under the PRJNA1452474 and PRJNA1510174 in the NCBI database.

## SUPPLEMENTAL MATERIAL

Figure S1 spectrum.01502-26-s0001.pdf Genomic comparison of plasmids identified in this study.

Figure S2 spectrum.01502-26-s0002.pdf Global characteristics of the NTS ST34 data set (*n* = 691).

Figure S3 spectrum.01502-26-s0003.pdf Temporal and demographic analysis of the ST34 lineage.

Supplemental material spectrum.01502-26-s0004.docx Tables S1, S3, and S4; legends for Fig. S1 to S3.

Table S2 spectrum.01502-26-s0005.xlsx Metadata of all ST34 NTS genomes.

Table S5 spectrum.01502-26-s0006.xlsx Virulence profiles of 77 NTS isolates.

Table S6 spectrum.01502-26-s0007.xlsx Clustering of study isolates based on a cgSNP distance threshold of <10 SNPs.

OPEN PEER REVIEW reviewer-comments.pdf An accounting of the reviewer comments and feedback.

ASM does not own the copyrights to Supplemental Material that may be linked to, or accessed through, an article. The authors have granted ASM a non-exclusive, world-wide license to publish the Supplemental Material files. Please contact the corresponding author directly for reuse.
