# Supplementary material for: Genomic epidemiology and antimicrobial resistance of non-typhoidal Salmonella in children in Hainan Province, China
Source: Microbiol Spectr. 2026 Sep 23;14(11):e01502-26. doi: 10.1128/spectrum.01502-26 (PMC13729223; doi:10.1128/spectrum.01502-26)
Supplement: Supplementary file 1 [file reviewer-comments.pdf]

ASM | Microbiology Spectrum

Genomic Epidemiology and Antimicrobial Resistance of Non-typhoidal *Salmonella* in Children in Hainan Province, China

Huaiyu Jia, Tao Chen, Xiaozhuang Zhang, Guozhen Tian, and Min Wang

Corresponding Author(s): Min Wang, Hainan Women and Children's Medical Center

Review Timeline:

Submission Date:	May 2, 2026
Editorial Decision:	July 6, 2026
Revision Received:	August 15, 2026
Accepted:	August 21, 2026

Editor: Sandeep Tamber

Reviewer(s): Disclosure of reviewer identity is with reference to reviewer comments included in decision letter(s). The following individuals involved in review of your submission have agreed to reveal their identity: Min Yue (Reviewer #2)

Transaction Report:

(Note: With the exception of the correction of typographical or spelling errors that could be a source of ambiguity, letters and reports are not edited. The original formatting of letters and referee reports may not be reflected in this compilation.)

DOI: https://doi.org/10.1128/spectrum.01502-26

Re: Spectrum01502-26 (**Genomic Epidemiology and Antimicrobial Resistance of Non-typhoidal*Salmonella* in Children in Hainan Province, China**)

Dear Prof. Min Wang:

Thank you for the privilege of reviewing your work. Below you will find my comments, instructions from the Spectrum editorial office, and the reviewer comments.

Both reviewers offer excellent suggestions to improve the context of the study the data interpretation. Please follow each of their suggestions and indicate in your response how each was addressed. Please verify all sequence data is deposited and include the accession numbers for any sequences that may not be a part of the Bioproject PRJNA1452474.

Please return the manuscript within 60 days; if you cannot complete the modification within this time period, please contact me. If you do not wish to modify the manuscript and prefer to submit it to another journal, notify me immediately so that the manuscript may be formally withdrawn from consideration by Spectrum.

Revision Guidelines
To submit your modified manuscript, log into the submission site at https://spectrum.msubmit.net/cgi-bin/main.plex. Go to Author Tasks and click the appropriate manuscript title to begin. The information you entered when you first submitted the paper will be displayed; update this as necessary. Note the following requirements:

• Upload point-by-point responses to the issues raised by the reviewers in a file named "Response to Reviewers," NOT in your cover letter.
• Upload a compare copy of the manuscript (without figures) as a "Marked-Up Manuscript" file.
• Upload a clean .DOC/.DOCX version of the revised manuscript and remove the previous version.
• Each figure must be uploaded as a separate, editable, high-resolution file (TIFF or EPS preferred), and any multipanel figures must be assembled into one file.
• Any supplemental material intended for posting by ASM should be uploaded with their legends separate from the main manuscript. You can combine all supplemental material into one file (preferred) or split it into a maximum of 10 files with all associated legends included.

For complete guidelines on revision requirements, see our Submission and Review Process webpage. Submission of a paper that does not conform to guidelines may delay acceptance of your manuscript.

Data availability: ASM policy requires that data be available to the public upon online posting of the article, so please verify all links to sequence records, if present, and make sure that each number retrieves the full record of the data. If a new accession number is not linked or a link is broken, provide Spectrum production staff with the correct URL for the record. If the accession numbers for new data are not publicly accessible before the expected online posting of the article, publication may be delayed; please contact production staff (Spectrum@asmusa.org) immediately with the expected release date.

Publication Fees: For information on publication fees and which article types are subject to charges, visit ourwebsite. If your manuscript is accepted for publication and any fees apply, you will be contacted separately about payment during the production process; please follow the instructions in that e-mail. Arrangements for payment must be made before your article is published.

ASM Membership: Corresponding authors may join or renew ASM membership to obtain discounts on publication fees. Need to upgrade your membership level? Please contact Customer Service at Service@asmusa.org.

The ASM Journals program strives for constant improvement in our submission and publication process. Please tell us how we can improve your experience by taking this quick Author Survey.

Thank you for submitting your paper to Spectrum.

Sincerely,
Sandeep Tamber
Editor
Microbiology Spectrum

Reviewer #1 (Comments for the Author):

This study investigated molecular typing, antimicrobial resistance and virulence characteristics of non-typhoidal Salmonella. The research design is reasonable overall. However, multiple issues regarding academic writing, data presentation, figure quality,

experimental rationality and information completeness need to be addressed thoroughly. Revision is required before further consideration. Detailed comments are listed below:
1. Inappropriate description and logical errors
Lines 202-204: The statement about MLST typing is not rigorous. MLST targets conserved housekeeping genes that are responsible for basic physiological metabolism of bacteria, rather than resistance or virulence genes. Accordingly, isolates with the same sequence type (ST) do not necessarily have identical resistance phenotypes or genotypes. Please revise this part.
2. Incomplete data and information annotation
1) Lines 161-163: For all percentage data, please mark the number of positive isolates / total isolates to keep consistent with the preceding text.
2) Lines 223-238: The original strain IDs are missing in the text. Please clearly list the strain number, corresponding virulence genes and serotype of each isolate.
3) Lines 207-214: The manuscript describes the resistance gene NDM-5, its flanking mobile genetic elements and plasmid location, but no supporting data are provided. Relevant evidence should be supplemented.
3. Questions on experimental methods
Third-generation long-read sequencing was not performed on strain S56. Please elaborate on the methods used to determine the location of resistance genes such as NDM-5, and explain the basis for the relevant results.
4. Figures and legends
1) Lines 189-190: The textual description is inconsistent with the results shown in Figure 1. Please rephrase the content to accurately reflect the figure data.
2) Figures S1A and S1B have low resolution. Please replace them with high-definition images.
3) Figure S1C: The figure legend does not explain the meaning of different modules. Please add detailed annotations by referring to relevant published literature.
5. Dataset deposition
This study includes second-generation sequencing data of 77 non-typhoidal Salmonella isolates, as well as third-generation sequencing data of strains S6, S20, S55 and S76. Please deposit these large datasets into a public repository.

Reviewer #2 (Comments for the Author):

Single-center retrospective WGS study of 77 pediatric NTS isolates (Hainan Women and Children's Medical Center, Apr 2023-May 2024). Integrates AST (VITEK + disk), Illumina (+ selective hybrid Nanopore) sequencing, serotyping (SeqSero2/SISTR), MLST, pangenome/phylogenetics (Roary/RAxML/IQ-TREE), AMR gene/plasmid profiling (AMRFinderPlus/ABRicate), and BEAST phylodynamics on global ST34 collection (PATRIC). Key claims includes 10.4% invasive rate (blood), strong predominance of MDR monophasic 1,4,[5],12:i:- ST34 (40.3%), emergence of blaNDM-5 (IncHI2A plasmids, 3 isolates) and tet(X4) (multi-replicon plasmid), China-specific ST34 clade B clonal expansion with limited international intermingling, and call for One Health surveillance in tropical regions.

This is a timely, well-executed regional baseline study that addresses a clear gap in pediatric NTS molecular epidemiology in China's only tropical island province. Strengths include the integrated genomic-phenotypic approach, high-quality hybrid plasmid reconstructions for high-risk elements (NDM-5 and tet(X4)), informative visualizations (resistance heatmap, core-genome trees with metadata tracks, global ST34 phylogeography), and open data (PRJNA1452474). Findings align with and extend the rapidly evolving literature on ST34 monophasic expansion, plasmid-mediated last-line resistance emergence in NTS, and heightened vulnerability of young children in tropical/LMIC settings. The work is complementary to recent national and global efforts (e.g., Nat Commun global AMR atlas in Salmonella, iNTS meta-analyses in China, and ST34-specific genomic studies). However, the key citations should be included (see later).

Here are the Major Comments:
1. The study is almost entirely descriptive. Add 95% confidence intervals for all key proportions (invasive rate 8/77, resistance % by serotype/ST, MDR rates). Perform and report appropriate statistical tests: χ^2 or Fisher's exact for serotype-ST linkage (stated as "strongly associated" but untested), resistance differences across dominant clones, age group vs. invasive infection, or season vs. detection. For the cgSNP <30 "recent clonal expansion" claim in clade B, either justify the threshold with literature/sensitivity analysis or use a standard recent-transmission cutoff (commonly {less than or equal to}5-10 SNPs) plus temporal clustering. Small n for rare serotypes (n=2-5) makes % resistance unreliable without CIs or caveats. Logistic regression could explore predictors of invasiveness or MDR if covariates allow.

2. TempEst root-to-tip regression is mentioned but no correlation coefficient, R^2, or p-value is reported-essential to demonstrate temporal signal before BEAST. Clarify the exact demographic model ("coalescent tree prior with constant population size and Bayesian skyline" reads as potentially two priors; specify if skyline plot was used for variable population size). Report ESS values for all key parameters (not just >200 overall), clock rate, TMRCA estimates with 95% HPD, and convergence diagnostics (Tracer). Justify random subsampling to 139 China ST34 genomes and test sensitivity. The 1997 China MRCA and post-2017 decline are interesting-compare explicitly to published ST34 emergence timelines (Europe/Asia ~1980s-1990s, with regional waves).

3. The 10.4% invasive (blood) rate in young children (85.7% aged 0-3 years) is highlighted as elevated. Quantitatively

benchmark against recent Chinese pediatric hospital series (e.g., Guangzhou, Hangzhou, Fuzhou, Ningbo) and national iNTS meta-analyses (children/infants disproportionately affected; overall China iNTS CFR ~8.6%). Discuss potential upward bias from single tertiary referral center (more severe cases). The summer-autumn peak aligns with national data, but the tropical year-round warm climate hypothesis for enhanced survival/transmission needs stronger support or qualification. Male predominance (55.8%) and gastro ward dominance are consistent with literature but should be statistically tested vs. expected.

4. The China-specific clade B expansion with zoonotic (pig-human) intermingling and limited international clustering is well shown. Explicitly link to recent work on ST34 evolutionary trajectory, including sequential gene loss signatures that may promote pandemic spread, prophage/Fim/bcsG dynamics, and host-adaptation. Screen or discuss whether Hainan isolates carry the same signatures. The tet(X4) plasmid similarity to E. coli/Morganella and NDM-5 insertion into conserved IncHI2A backbones strongly support inter-species HGT risk-expand discussion with recent Chinese ST34 reports documenting identical elements. Emphasize implications for last-resort agents in pediatrics and the need for integrated human-animal-food surveillance in tropical island settings (link to national AMR plans and infant food safety initiatives).

5. The hybrid-assembly plasmid work (pS6-NDM-5, pS55-NDM-5, tet(X4) mosaic plasmid) is a highlight. Add conjugation/transmission data if available, or discuss temperature-dependent efficiency (recent work shows higher at 26{degree sign}C). Position these as early signals of carbapenem and tigecycline resistance emergence in NTS ST34 in tropical China-timely given parallel 2026 reports from Zhejiang. Recommend authors discuss surveillance thresholds for these mobile elements.

6. Confirm exact CLSI (M100 36th ed. 2026) and EUCAST (v16.1 2026) versions/editions used. For the four hybrid assemblies (S6, S20, S55, S76), state selection criteria (carbapenem/tigecycline resistant?) and report assembly QC (N50, BUSCO/completeness). PATRIC 660 ST34 genomes-any quality filtering or metadata completeness checks? Roary/RAxML parameters for the all-isolate tree should be explicit. Serotype-ST concordance is biologically expected but still merits a supplementary contingency table or test.

7. Table 1 season data is confusing (ratios appear inconsistent with n=77 total; footnote on "detection rate in fecal culture specimens" needs clarification of denominator). Figure 1 resistance heatmap is excellent-consider adding statistical annotations if tests performed. Figure 4 global ST34 tree is visually strong; ensure metadata rings (host, region, year, serotype) are fully legible. Legends are generally clear. Supplementary plasmid comparisons (Figure S1) are referenced but not described in main text-briefly summarize key backbone/insertion findings.

8. Limitations section is honest (single center, no concurrent animal/food isolates, small n for rares) but could be more quantitative (statistical power). Strengthen the One Health call with concrete, actionable recommendations (e.g., sentinel surveillance sites linking pediatric hospitals to veterinary/food labs in Hainan/Guangdong; integration with existing national systems). Future directions (longitudinal WGS monitoring, source attribution, functional plasmid/virulence studies) are appropriate.

9. Key references that are missing in the current context.
The first patient-pathogen study of invasive NTS: Nature Medicine 31(7):2325-2334. doi:10.1038/s41591-025-03644-4.
One Health study of iNTS origin and further confine the human-centered evolution: National Science Review nwag275. doi: 10.1093/nsr/nwag275
Updated genomic evolution of iNTS ST313, with high relevance to Typhimurium ST34: Nature Communications 17:3693. doi: 10.1038/s41467-026-70196-7.
The first China-wide iNTS study: Journal of Global Health 16:04008. doi: 10.7189/jogh.16.04008.

ST34 global and local picture, particularly revelant in China.
Frontiers in Cellular and Infection Microbiology 15:1490183. doi: 10.3389/fcimb.2025.1490183.
And additional review in the field.

The key problem of this study is lack of contextual knowledge, please include this and integrate additional information for a meaningful discussion

minor points:
Typos/editing: "Abstrast" (Abstract). Minor inconsistencies in email formatting. Occasional awkward phrasing or long sentences in Introduction/Discussion-light copy-editing recommended. "10.4% (8/77)" vs. "10.39%" standardize. In Results, complete ST list or consistently reference Table S1.

Abstract/Importance: Both are strong summaries. Importance could be slightly tightened; ensure it does not overstate "first local molecular epidemiological baseline" without acknowledging any prior non-genomic Hainan data.

Data availability/ethics/funding: please doubel check the avilablibity of the data, and etc. (PRJNA1452474, ethics approval stated, funding acknowledged)

This study investigated molecular typing, antimicrobial resistance and virulence characteristics of non-typhoidal Salmonella. The research design is reasonable overall. However, multiple issues regarding academic writing, data presentation, figure quality, experimental rationality and information completeness need to be addressed thoroughly. Revision is required before further consideration. Detailed comments are listed below:

1. Inappropriate description and logical errors

Lines 202–204: The statement about MLST typing is not rigorous. MLST targets conserved housekeeping genes that are responsible for basic physiological metabolism of bacteria, rather than resistance or virulence genes. Accordingly, isolates with the same sequence type (ST) do not necessarily have identical resistance phenotypes or genotypes. Please revise this part.

2. Incomplete data and information annotation

1) Lines 161–163: For all percentage data, please mark the number of positive isolates / total isolates to keep consistent with the preceding text.

2) Lines 223–238: The original strain IDs are missing in the text. Please clearly list the strain number, corresponding virulence genes and serotype of each isolate.

3) Lines 207–214: The manuscript describes the resistance gene NDM-5, its flanking mobile genetic elements and plasmid location, but no supporting data are provided. Relevant evidence should be supplemented.

3. Questions on experimental methods

Third-generation long-read sequencing was not performed on strain S56. Please elaborate on the methods used to determine the location of resistance genes such as NDM-5, and explain the basis for the relevant results.

4. Figures and legends

1) Lines 189–190: The textual description is inconsistent with the results shown in

Figure 1. Please rephrase the content to accurately reflect the figure data.

2) Figures S1A and S1B have low resolution. Please replace them with high-definition images.

3) Figure S1C: The figure legend does not explain the meaning of different modules. Please add detailed annotations by referring to relevant published literature.

5. Dataset deposition

This study includes second-generation sequencing data of 77 non-typhoidal Salmonella isolates, as well as third-generation sequencing data of strains S6, S20, S55 and S76. Please deposit these large datasets into a public repository.

Dear Dr. Tamber and Reviewers,

We are grateful to you and the two reviewers for the careful evaluation of our manuscript. The comments have substantially improved the statistical rigor, methodological transparency, and contextual depth of our work. In the revised manuscript, we have added 95% confidence intervals and statistical tests for all key estimates, expanded the reporting of the phylodynamic analyses, benchmarked our findings against recent national and global studies, incorporated all recommended references, revised the figures and tables, and verified the deposition of all sequencing data. Below is our point-by-point response.

Sincerely,

Min Wang

On behalf of all authors

Email: wangminsuer@163.com

Response to the Editor

Comment: Please verify all sequence data is deposited and include the accession numbers for any sequences that may not be a part of the Bioproject PRJNA1452474.

Response: We have verified that all sequencing data are publicly accessible. The draft assembly data for the 77 *Salmonella* isolates have been deposited under BioProject accession number PRJNA1452474. The hybrid assembly of strains S6, S20, S55, and S76 have been deposited under BioProject accession number PRJNA1510174. We have updated the "Data Availability" in the revised manuscript to include these accession numbers.

Changes: **Data Availability** (page 21, lines 454-456).

Response to Reviewer 1

We thank Reviewer 1 for the detailed comments, which have improved the rigor of our data presentation and writing.

Comment 1: Inappropriate description and logical errors. Lines 202-204: The statement about MLST typing is not rigorous. MLST targets conserved housekeeping genes that are responsible for basic physiological metabolism of bacteria, rather than resistance or virulence genes. Accordingly, isolates with the same sequence type (ST) do not necessarily have identical resistance phenotypes or genotypes. Please revise this part.

Response: We agree that MLST targets conserved housekeeping genes responsible for essential cellular functions rather than resistance or virulence genes, and that isolates

sharing the same ST do not necessarily exhibit identical resistance phenotypes or genotypes. We have revised the relevant statements so that the relationship between STs and resistance is presented strictly as an observed association within this collection rather than as an intrinsic property of the ST. We have checked the entire manuscript to ensure that no wording implies that ST determines resistance or virulence.

Changes: Results (page 11, lines 229-231).

Comment 2: Incomplete data and information annotation. **1)** Lines 161-163: For all percentage data, please mark the number of positive isolates / total isolates to keep consistent with the preceding text. **2)** Lines 223-238: The original strain IDs are missing in the text. Please clearly list the strain number, corresponding virulence genes and serotype of each isolate. **3)** Lines 207-214: The manuscript describes the resistance gene NDM-5, its flanking mobile genetic elements and plasmid location, but no supporting data are provided. Relevant evidence should be supplemented.

Response: **1)** The number of positive or resistant isolates relative to the total number tested has been added for all percentage data throughout the Results. **2)** We have added Table S5 to the Supplementary Materials, which provides a comprehensive list of the strain numbers, corresponding virulence genes, and serotypes for each isolate. The text now references this table. **3)** We have added a description of the plasmid backbone and insertion site comparisons in the revised manuscript: Comparative analysis showed that the plasmids from S55 and S6 shared an identical backbone with high similarity (>90% coverage, 100% identity) to p3M-23 (China) and pZ1323SSL0063-1 (South Korea)

(Figure S1A). However, the latter two lacked the $bla_{\text{NDM-5}}$-carrying region, which is flanked by mobile genetic elements (*IS26*-*IS3000*-*IS5*-$bla_{\text{NDM-5}}$-*IS26*). suggesting acquisition of $bla_{\text{NDM-5}}$ via insertion into a conserved backbone (Figure S1B).

Changes: **1)** Results (page 9, lines 177-187); **2)** Results (page 12, lines 253-260), Table S5; **3)** Results (page 12, lines 239-244).

Comment 3: Questions on experimental methods. Third-generation long-read sequencing was not performed on strain S56. Please elaborate on the methods used to determine the location of resistance genes such as NDM-5, and explain the basis for the relevant results.

Response: We thank the reviewer for this keen observation and for pointing out the lack of clarity regarding the sequencing methods for strain S56. We apologize for the oversight in our original description. To clarify, strains S55 and S6 were assembled using a hybrid approach combining Illumina short-reads and Oxford Nanopore long-reads to obtain complete genomes. However, strain S56 was sequenced using short-reads only. To determine the location of the blaNDM-5 gene in S56, we utilized Mob-suite to reconstruct and predict plasmid sequences from the short-read data. The analysis predicted that the $bla_{\text{NDM-5}}$ gene in S56 is located on an IncHI2A plasmid, which is the same as the complete plasmids identified in S55 and S6. We have revised the Methods section to specify the sequencing strategies and updated the Results section to clearly describe the identification method for the S56 plasmid.

Changes: Materials and Methods (page 6, lines 127-130); Results (page 11, lines 232-

244).

Comment 4: Figures and legends. **1)** Lines 189-190: The textual description is inconsistent with the results shown in Figure 1. Please rephrase the content to accurately reflect the figure data. **2)** Figures S1A and S1B have low resolution. Please replace them with high-definition images. **3)** Figure S1C: The figure legend does not explain the meaning of different modules. Please add detailed annotations by referring to relevant published literature.

Response: **1)** We apologize for the inconsistency. We have cross-checked the text against Figure 1 and revised the description to accurately reflect the figure data. **2)** We have replaced Figures S1A and S1B with vector graphics (PDF format). Theoretically, these images are resolution-independent and should remain clear at any magnification. The blurriness observed might be due to the compression or rendering process of the submission system's PDF viewer. We kindly suggest the reviewer download the original PDF file to view the high-definition images. We have also re-checked the files to ensure they meet the journal's requirements. **3)** We apologize for the lack of clarity in the original legend. In accordance with your suggestion, we have revised the legend for Figure S1 to provide detailed annotations for each module.

Changes: **1)** Results (page 10, lines 209-217), Figure 1 and its legend; **2)** Figures S1A and S1B; **3)** Figure S1 legend.

Comment 5: Dataset deposition. This study includes second-generation sequencing

data of 77 non-typhoidal Salmonella isolates, as well as third-generation sequencing data of strains S6, S20, S55 and S76. Please deposit these large datasets into a public repository.

Response: We sincerely thank the reviewer for this essential reminder regarding data transparency and accessibility. We fully agree that depositing the data into a public repository is crucial. The draft assembly data for the 77 *Salmonella* isolates have been deposited under BioProject accession number PRJNA1452474. The hybrid assembly of strains S6, S20, S55, and S76 have been deposited under BioProject accession number PRJNA1510174. We have updated the "Data Availability" in the revised manuscript to include these accession numbers.

Changes: Data Availability (page 21, lines 454-456).

Response to Reviewer 2

We thank Reviewer 2 for the positive assessment and for the constructive suggestions, which have substantially strengthened the statistical rigor and contextual framing of our study.

Comment 1: The study is almost entirely descriptive. **1)** Add 95% confidence intervals for all key proportions (invasive rate 8/77, resistance % by serotype/ST, MDR rates). **2)** Perform and report appropriate statistical tests: χ^2 or Fisher's exact for serotype-ST linkage (stated as "strongly associated" but untested), resistance differences across dominant clones, age group vs. invasive infection, or season vs. detection. **3)** For the cgSNP <30 "recent clonal expansion" claim in clade B, either justify the threshold with

literature/sensitivity analysis or use a standard recent-transmission cutoff (commonly {less than or equal to}5-10 SNPs) plus temporal clustering. **4)** Small n for rare serotypes (n=2-5) makes % resistance unreliable without CIs or caveats. Logistic regression could explore predictors of invasiveness or MDR if covariates allow.

Response: **1)** Ninety-five percent confidence intervals (Clopper-Pearson exact method) have been added for all key proportions. The complete set of estimates with CIs is reported in the Results and in Table 2. **2)** All requested comparisons involve categorical variables (e.g., serotype *vs.* ST, clone *vs.* resistance phenotype, age group *vs.* infection status, season *vs.* detection). We have therefore applied χ^2 tests or Fisher's exact tests as appropriate. The corresponding *P* values have been added to the revised Tables. **3)** We agree that the original use of a cgSNP distance of <30 SNPs as evidence of recent clonal expansion was insufficiently justified. We therefore re-evaluated the closely related isolates using a more stringent cgSNP threshold of <10 SNPs and incorporated sampling dates to assess temporal clustering. The revised analysis identified four closely related clusters of 16 study isolates separated by <10 cgSNPs and collected within 8 months, supporting recent clonal expansion. We have therefore revised the corresponding statement in the Results to avoid interpreting the <30-SNP threshold as a definitive transmission cutoff. **4)** Given the small sample sizes of rare serotypes (n=2-5), we have added Clopper-Pearson exact 95% confidence intervals to all resistance proportions and added a footnote stating that estimates for these subgroups are imprecise and should be interpreted with caution. We opted not to perform multivariable logistic regression because the number of events per variable would be

insufficient for reliable estimation, particularly within rare serotypes and specific resistance phenotypes. These revisions are reflected in the updated Results, Tables, and footnotes.

Changes: **1)** Abstract (page 2, lines 29-38), Results (page 9, lines 188-201; page 10, lines 209-215), Table 2; **2)** Materials and Methods (page 8, lines 167-173), Results (page 9, lines 176-187), Table 1; **3)** Results (page 13, lines 276-279); **4)** Results (page 10, lines 215-217), Figure 1 and its legend, Table S3.

Comment 2: **1)** TempEst root-to-tip regression is mentioned but no correlation coefficient, R^2, or p-value is reported-essential to demonstrate temporal signal before BEAST. **2)** Clarify the exact demographic model ("coalescent tree prior with constant population size and Bayesian skyline" reads as potentially two priors; specify if skyline plot was used for variable population size). Report ESS values for all key parameters (not just >200 overall), clock rate, TMRCA estimates with 95% HPD, and convergence diagnostics (Tracer). **3)** Justify random subsampling to 139 China ST34 genomes and test sensitivity. **4)** The 1997 China MRCA and post-2017 decline are interesting-compare explicitly to published ST34 emergence timelines (Europe/Asia ~1980s-1990s, with regional waves).

Response: We thank the reviewer for this crucial observation. **1)** We have now included the correlation coefficient and *P*-values for the root-to-tip regression in the revised manuscript (correlation coefficient = 0.6028, $P < 0.0001$). **2)** Additionally, we clarified that the Bayesian Skyline model was used (not constant size) and have added a

description reporting ESS values for key parameters, including the clock rate (ESS = 237) and TMRCA with 95% HPD (ESS = 264). **3)** A total of 139 genomes were selected using a stratified sampling strategy to ensure the representation of Hainan isolates and a balanced temporal distribution across the study period, while maintaining computational feasibility for Bayesian analysis. We acknowledge that subsampling can influence parameter estimates. As suggested, we have added a statement in the Discussion section explicitly recognizing this as a limitation and discussing the potential impact on our TMRCA estimates. **4)** We acknowledge that the global ST34 lineage (particularly the European clone) emerged earlier, around the 1980s. Our estimated TMRCA of 1997 for the Chinese lineage is indeed later. We discuss this in the context of regional waves, noting that our result mirrors recent large-scale studies in Southeast Asia, which estimated a regional MRCA of 1995 (95% HPD: 1992–1998). This suggests a synchronized regional emergence distinct from the earlier European expansion. We have elaborated on the Post-2017 decline in effective population size observed in our Bayesian Skyline Plot. We attribute this potentially to the stringent national action plans implemented in China around 2016–2017 (e.g., the ban on colistin as a growth promoter), which may have reduced the selective advantage of these multidrug-resistant strains. We also explicitly mention the possibility of sampling bias as a contributing factor.

Changes: **1)** Results (page 14, lines 283-285), Figure S3; **2)** Materials and Methods (page 8, lines 158-162), Results (page 14, lines 285-289); **3)** Materials and Methods (page 8, lines 153-155), Discussion (page 20, lines 421-431); **4)** Discussion (page 18,

lines 385-396).

Comment 3: **1)** The 10.4% invasive (blood) rate in young children (85.7% aged 0-3 years) is highlighted as elevated. Quantitatively benchmark against recent Chinese pediatric hospital series (e.g., Guangzhou, Hangzhou, Fuzhou, Ningbo) and national iNTS meta-analyses (children/infants disproportionately affected; overall China iNTS CFR ~8.6%). Discuss potential upward bias from single tertiary referral center (more severe cases). **2)** The summer-autumn peak aligns with national data, but the tropical year-round warm climate hypothesis for enhanced survival/transmission needs stronger support or qualification. **3)** Male predominance (55.8%) and gastro ward dominance are consistent with literature but should be statistically tested vs. expected.

Response: **1)** Our 10.4% invasive infection rate is among the higher rates reported in recent Chinese pediatric hospital series. We explicitly acknowledge that, as the province's sole tertiary pediatric referral centre, our hospital is likely to over-represent severe cases, which may inflate the observed invasive proportion. **2)** We have tempered the tropical climate hypothesis in the revised Discussion: While year-round warm temperatures may facilitate bacterial survival and transmission, direct supporting environmental or food-chain evidence is lacking. **3)** We added formal statistical testing: the male proportion (55.8%) was compared against the expected 50% using an exact binomial test, and goodness-of-fit χ^2 tests were used to compare ward and age group distributions against uniform distributions. Results are reported in table footnotes.

Changes: **1)** Discussion (page 14, lines 300-301), (page 20, lines 415-418); **2)**

Discussion (page 14, lines 302-303); **3)** Materials and Methods (page 8, lines 170-173), Table 1.

Comment 4: **1)**The China-specific clade B expansion with zoonotic (pig-human) intermingling and limited international clustering is well shown. Explicitly link to recent work on ST34 evolutionary trajectory, including sequential gene loss signatures that may promote pandemic spread, prophage/Fim/bcsG dynamics, and host-adaptation. Screen or discuss whether Hainan isolates carry the same signatures. **2)**The tet(X4) plasmid similarity to E. coli/Morganella and NDM-5 insertion into conserved IncHI2A backbones strongly support inter-species HGT risk-expand discussion with recent Chinese ST34 reports documenting identical elements. **3)**Emphasize implications for last-resort agents in pediatrics and the need for integrated human-animal-food surveillance in tropical island settings (link to national AMR plans and infant food safety initiatives).

Response: **1)** We appreciate this suggestion and have added a new paragraph in the Discussion linking our findings to recent work on the evolutionary trajectory of ST34. We now discuss: (i) the stepwise gene loss events involving prophage regions (Gifsy-1, HP1) and *IS26*-mediated microevolution around the *fljAB* locus that have been associated with the generation of clonally expanding sublineages; (ii) the acquisition of prophages mTmV carrying *sopE* and *perC* genes that facilitate epithelial cell invasion and gut niche adaptation; (iii) SGI-4 and Tn21 transposon-mediated resistance to heavy metals and antibiotics supports its persistence in intensive livestock farming

environments; (iv) and the progressive degradation of virulence genes (such as *cspC*) reflects an adaptive shift from high virulence towards a high-transmission strategy. **2)** We fully agree with the reviewer and have substantially expanded the Discussion to address the inter-species HGT risk. We now explicitly discuss: (i) the similarity between the *tet(X4)*-harboring IncFIA/HI1A/HI1B mosaic plasmid in our isolates and *tet(X4)*-bearing plasmids among *E. coli* and *Morganella* spp. in China; (ii) the insertion of *bla*$_{\text{NDM-5}}$ into a conserved IncHI2A backbone, mirroring the genetic architecture of NDM-5-carrying IncHI2/IncHI2A plasmids recently reported in *Salmonella* and *E. coli* from food animals in China. **3)** We have added new paragraphs in the Discussion addressing: (i) the clinical implications for pediatric populations, emphasizing that the occurrence of *bla*$_{\text{NDM-5}}$ and *tet(X4)* on plasmids within ST34 compromises both carbapenems and tigecycline, critical last-resort agents for pediatric salmonellosis; and (ii) the need for integrated human-animal-food surveillance programs in tropical island settings such as Hainan, aligned with China's National Action Plan on Containing Antimicrobial Resistance (2022–2025). We also emphasize the vulnerability of infants and young children to foodborne salmonellosis and the importance of enhanced surveillance of NTS in infant food products.

Changes: **1)** Discussion (page 17, lines 361-384); **2)** Discussion (page 15, lines 322-339); **3)** Discussion (page 17, lines 351-359), (page 19, lines 406-414).

Comment 5: **1)** The hybrid-assembly plasmid work (pS6-NDM-5, pS55-NDM-5, tet(X4) mosaic plasmid) is a highlight. Add conjugation/transmission data if available,

or discuss temperature-dependent efficiency (recent work shows higher at 26{degree sign}C). **2)** Position these as early signals of carbapenem and tigecycline resistance emergence in NTS ST34 in tropical China-timely given parallel 2026 reports from Zhejiang. **3)** Recommend authors discuss surveillance thresholds for these mobile elements.

Response: **1)** We appreciate this insightful suggestion. In the current study, experimental conjugation assays were not performed; however, we have added a dedicated discussion on the temperature-dependent conjugative transfer efficiency of the identified plasmids. We note that certain plasmid incompatibility groups (like IncHI1-type plasmids) exhibit thermosensitive conjugative transfer with markedly higher frequencies at ambient temperatures (< 30°C) compared to 37°C. Given the tropical climate of Hainan, where our isolates were collected, such mechanisms may differentially influence plasmid transfer dynamics across ecological niches, potentially enhancing environmental dissemination in soil and water reservoirs. We have also highlighted the need for future temperature-gradient conjugation experiments to quantify the transmission potential of these plasmids under both environmental and host-associated conditions. **2)** We fully agree with the reviewer and have revised the Discussion to explicitly position our findings as an early warning signal of the emergence of carbapenem and tigecycline resistance in NTS ST34 in tropical China. We now emphasize that the occurrence of $\mathit{bla}_{\mathrm{NDM\text{-}5}}$ and *tet*(X4) on plasmids within this high-risk clone effectively compromises two of the last-resort therapeutic options for salmonellosis. Furthermore, we have incorporated references to underscore that the

emergence of carbapenem-resistant NTS ST34 is not an isolated event but a growing regional trend across southern China. **3)** We have added a new paragraph in the Discussion addressing surveillance considerations for high-risk mobile genetic elements. Specifically, we recommend that surveillance programs in China and similar tropical regions incorporate molecular screening thresholds for *bla*NDM-carrying plasmids and *tet*(X4)-harboring plasmids, particularly when identified in ST34 or other internationally disseminated clones. We propose that predefined alert thresholds, such as detection of these plasmids exceeding a defined proportion of sentinel surveillance isolates, or their identification in food animal or environmental reservoirs, could facilitate early intervention before widespread clonal expansion occurs.

Changes: **1)** Discussion (page 16, lines 339-350); **2)** Discussion (page 17, lines 351-359); **3)** Discussion (page 19, lines 397-406).

Comment 6: **1)** Confirm exact CLSI (M100 36th ed. 2026) and EUCAST (v16.1 2026) versions/editions used. **2)** For the four hybrid assemblies (S6, S20, S55, S76), state selection criteria (carbapenem/tigecycline resistant?) and report assembly QC (N50, BUSCO/completeness). **3)** PATRIC 660 ST34 genomes-any quality filtering or metadata completeness checks? Roary/RAxML parameters for the all-isolate tree should be explicit. **4)** Serotype-ST concordance is biologically expected but still merits a supplementary contingency table or test.

Response: **1)** We have confirmed that results were interpreted according to CLSI M100 36th edition (2026) and EUCAST 2026 v16.0, and the Methods now cite the exact

editions. **2)** We thank the reviewer for this suggestion. The four isolates (S6, S20, S55, and S76) were selected for additional Oxford Nanopore MinION sequencing and hybrid assembly due to the presence of critical antimicrobial resistance determinants, specifically *bla*$_{\text{NDM-5}}$, *tet*$_{\text{(X4)}}$, or multiple copies of *bla*$_{\text{TEM-1}}$. Regarding assembly quality, the hybrid assemblies yielded high-quality, near-complete genomes. As shown in Table S1, the assemblies resulted in only 2 to 5 contigs per isolate, with N50 values ranging from 4.68 Mb to 5.04 Mb, indicating that the majority of the genome was assembled into single large contigs (likely representing the chromosome and plasmids). **3)** All 660 ST34 genomes retrieved from PATRIC were subjected to rigorous quality control. We used the EvalG tool within the PATRIC pipeline to assess genome completeness and contamination. Genomes with completeness < 90% or contamination > 5% were excluded from further analysis. Additionally, we verified the completeness of key metadata fields (isolation source, collection year, and isolation country) and excluded 27 isolates with critical metadata missing. The final dataset used for analysis consisted of 660 high-quality genomes. Pan-genome analysis was performed using Roary v3.11.2 with default parameters. The maximum-likelihood phylogenetic tree was inferred from the core gene alignment with RAxML v8.2.12 using the GTRGAMMA substitution model (-m GTRGAMMA). Branch support was assessed with 100 rapid bootstrap replicates. **4)** Chi-square analysis of serotype-ST concordance has been added to the Methods and Results. Serotype and ST showed perfect one-to-one correspondence (Cramér's V = 1.000), with monophasic 1,4,[5],12:i:- (ST34) and Typhimurium (ST19) being the most prevalent serotypes (Table S4).

Changes: **1)** Materials and Methods (page 6, lines 117-120); **2)** Materials and Methods (page 6, lines 128-132); **3)** Materials and Methods (page 7, lines 135-146); **4)** Materials and Methods (page 8, lines 167-169), Results (page 11, lines 223-225).

Comment 7: **1)** Table 1 season data is confusing (ratios appear inconsistent with n=77 total; footnote on "detection rate in fecal culture specimens" needs clarification of denominator). **2)** Figure 1 resistance heatmap is excellent-consider adding statistical annotations if tests performed. **3)** Figure 4 global ST34 tree is visually strong; ensure metadata rings (host, region, year, serotype) are fully legible. Legends are generally clear. **4)** Supplementary plasmid comparisons (Figure S1) are referenced but not described in main text-briefly summarize key backbone/insertion findings.

Response: **1)** Table 1 has been revised with clarified denominators. The footnote now explicitly defines the detection rate as the number of NTS isolates from fecal culture divided by the total number of fecal culture specimens submitted during the corresponding period. **2)** To avoid overcrowding Figure 1, we have placed statistical comparisons of resistance rates among predominant serotypes in Table S3 (Fisher's exact tests), with the figure legend updated accordingly. **3)** Figure 4 has been regenerated with enlarged, fully legible metadata rings (host, region, year, and serotype). **4)** The Results now briefly summarize the key findings of Figure S1: Comparative analysis showed that the plasmids from S55 and S6 shared an identical backbone with high similarity (>90% coverage, 100% identity) to p3M-23 (China) and pZ1323SSL0063-1 (South Korea) (Figure S1A). However, the latter two lacked the

bla$_{NDM-5}$-carrying region, which is flanked by mobile genetic elements (*IS26*-*IS3000*-*IS5*-*bla*$_{NDM-5}$-*IS26*), suggesting acquisition of *bla*$_{NDM-5}$ via insertion into a conserved backbone (Figure S1B).

Changes: **1)** Table 1 (page 28-29); **2)** Figure 1' legend, Table S3; **3)** Figure 4; **4)** Results (page 12, lines 239-244).

Comment 8: Limitations section is honest (single center, no concurrent animal/food isolates, small n for rares) but could be more quantitative (statistical power). Strengthen the One Health call with concrete, actionable recommendations (e.g., sentinel surveillance sites linking pediatric hospitals to veterinary/food labs in Hainan/Guangdong; integration with existing national systems). Future directions (longitudinal WGS monitoring, source attribution, functional plasmid/virulence studies) are appropriate.

Response: The Limitations now state that: First, as a single provincial tertiary pediatric referral center receiving severe cases from across Hainan, our 77-isolate cohort may not fully represent the broader pediatric population, and the invasive infection rate (10.4%) may be overestimated owing to upward referral bias. Second, resistance estimates for rare serotypes (n=2-5) are imprecise owing to limited sample sizes. Third, the absence of concurrent food, animal, and environmental isolates prevented direct source attribution and transmission tracing. Finally, due to the high computational demand of Bayesian coalescent frameworks, we subsampled the dataset to 139 representative genomes. To mitigate potential sampling bias, we utilized a stratified

sampling approach that prioritized the inclusion of Hainan isolates and ensured a balanced temporal distribution of sequences throughout the study period. However, this subsampling strategy may not capture the full extent of the genetic diversity present in the broader population. Consequently, while our estimates for the TMRCA and population dynamics provide valuable insights into the ST34 lineage in this region, they should be interpreted with the understanding that larger-scale genomic surveillance might reveal finer sub-structures or slightly different emergence timelines. The One Health recommendations have been made concrete: (i) sentinel surveillance linking pediatric hospitals to veterinary and food-safety laboratories in Hainan and Guangdong; (ii) integration of NTS whole-genome sequencing into national AMR surveillance; (iii) targeted monitoring of blaNDM-5 and tet(X4)-carrying plasmids in the food chain, particularly poultry and pork production; and (iv) longitudinal tracking of resistance trends and plasmid epidemiology to guide empirical therapy.

Changes: Discussion (page 20, lines 415-437).

Comment 9: The first patient-pathogen study of invasive NTS: Nature Medicine 31(7):2325-2334. doi:10.1038/s41591-025-03644-4. One Health study of iNTS origin and further confine the human-centered evolution: National Science Review nwag275. doi: 10.1093/nsr/nwag275 Updated genomic evolution of iNTS ST313, with high relevance to Typhimurium ST34: Nature Communications 17:3693. doi: 10.1038/s41467-026-70196-7. The first China-wide iNTS study: Journal of Global Health 16:04008. doi: 10.7189/jogh.16.04008. ST34 global and local picture,

particularly revelant in China. Frontiers in Cellular and Infection Microbiology 15:1490183. doi: 10.3389/fcimb.2025.1490183. And additional review in the field. The key problem of this study is lack of contextual knowledge, please include this and integrate additional information for a meaningful discussion

Response: We thank the reviewer for these essential references, all of which have been incorporated.

Changes: References (page 22-26).

Comment 10: **1)** Typos/editing: "Abstrast" (Abstract). Minor inconsistencies in email formatting. Occasional awkward phrasing or long sentences in Introduction/Discussion-light copy-editing recommended. "10.4% (8/77)" vs. "10.39%" standardize. In Results, complete ST list or consistently reference Table S1. **2)** Abstract/Importance: Both are strong summaries. Importance could be slightly tightened; ensure it does not overstate "first local molecular epidemiological baseline" without acknowledging any prior non-genomic Hainan data. **3)** Data availability/ethics/funding: please doubel check the avilablibity of the data, and etc. (PRJNA1452474, ethics approval stated, funding acknowledged)

Response: **1)** The typographical error “Abstrast” has been corrected to “Abstract”; email formatting has been standardized; the manuscript has undergone light copy-editing, with long sentences in the Introduction and Discussion shortened; the invasive rate is now reported consistently as “10.4% (8/77)”; and the complete ST list is now consistently referenced to Table S4. **2)** The Importance section has been tightened. **3)**

We have double-checked data availability, the ethics approval statement, and the funding acknowledgment.

Changes: Throughout the manuscript.

Re: Spectrum01502-26R1 (**Genomic Epidemiology and Antimicrobial Resistance of Non-typhoidal *Salmonella* in Children in Hainan Province, China**)

Dear Prof. Min Wang:

Thank you for your thoughtful and thorough revisions.

Your manuscript has been accepted, and I am forwarding it to the ASM production staff for publication. Your paper will first be checked to make sure all elements meet the technical requirements. ASM staff will contact you if anything needs to be revised before copyediting and production can begin. Otherwise, you will be notified when your proofs are ready to be viewed.

Data Availability: ASM policy requires that data be available to the public upon online posting of the article, so please verify all links to sequence records, if present, and make sure that each number retrieves the full record of the data. If a new accession number is not linked or a link is broken, provide production staff with the correct URL for the record. If the accession numbers for new data are not publicly accessible before the expected online posting of the article, publication may be delayed; please contact ASM production staff immediately with the expected release date.

Publication Fees: For information on publication fees and which article types have charges, please visit our website. We have partnered with Copyright Clearance Center (CCC) to collect author charges. If fees apply to your paper, you will receive a message from no-reply@copyright.com with further instructions. For questions related to paying charges through RightsLink, please contact CCC at ASM_Support@copyright.com or toll free at +1-877-622-5543. CCC makes every attempt to respond to all emails within 24 hours.

ASM Membership: Corresponding authors may join or renew ASM membership to obtain discounts on publication fees. Need to upgrade your membership level? Please contact Customer Service at Service@asmusa.org.

Embargo Policy: A press release may be issued as soon as the manuscript is posted on the Spectrum Latest Articles webpage. The corresponding author will receive an email with the subject line "ASM Journals Author Services Notification" when the article is available online.

Cover Image Submissions: If you would like to submit a potential Cover Image, please email a file and a short legend to spectrum@asmusa.org. Please note that we can only consider images that (i) the authors created or own and (ii) have not been previously published. By submitting, you agree that the image can be used under the same terms as the published article. Image File requirements: TIF/EPS, 7.5 inches wide by 8.25 inches tall (at least 2,250 pixels wide by 2,475 pixels tall), minimum 300 dpi resolution (600 dpi preferred), RGB, and no figure elements, e.g., arrows or panel labels. The legend should be a short description of the image, 1-2 sentences recommended. Please use the ASM Journals Cover Image Sizing Tool (https://journals.asm.org/pb-assets/html/asm-cover-sizing-tool.html) to ensure that your proposed cover image meets our size requirements.

The ASM Journals program strives for constant improvement in our submission and publication process. Please tell us how we can improve your experience by taking this quick Author Survey.

Thank you for submitting your paper to Spectrum.

Sincerely,
Sandeep Tamber
Editor
Microbiology Spectrum
