# Supplementary material for: Genomic epidemiology and antimicrobial resistance of non-typhoidal Salmonella in children in Hainan Province, China
Source: Microbiol Spectr. 2026 Sep 23;14(11):e01502-26. doi: 10.1128/spectrum.01502-26 (PMC13729223; doi:10.1128/spectrum.01502-26)
Supplement: Supplementary file 2 [file spectrum.01502-26-s0001.pdf]

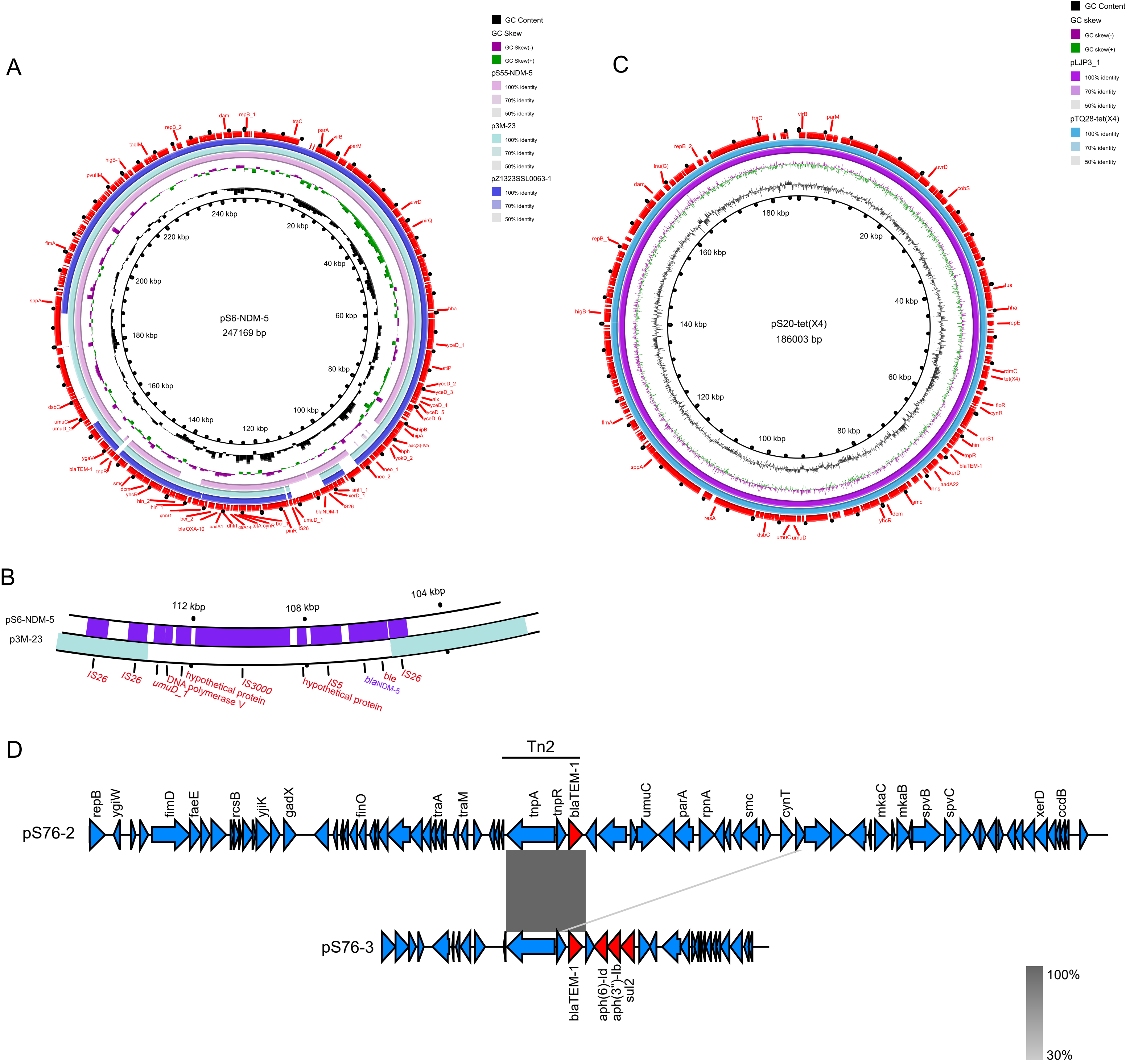

A
GC Content
GC Skew
GC Skew(-)
GC Skew(+)
pS55-NDM-5
100% identity
70% identity
50% identity
p3M-23
100% identity
70% identity
50% identity
pZ1323SSL0063-1
100% identity
70% identity
50% identity
pS6-NDM-5
247169 bp
20 kbp
40 kbp
60 kbp
80 kbp
100 kbp
120 kbp
140 kbp
160 kbp
180 kbp
200 kbp
220 kbp
240 kbp
dam
repB_1
traC
parA
virB
parM
repB_2
taqIM
higB-1
pvuIIM
uvrD
nirQ
flmA
hha
sppA
yceD_1
stiP
yceD_2
yceD_3
alx
yceD_4
yceD_5
yceD_6
dsbC
hipB
hipA
umuC
umuD_2
aac(3)-IVa
hph
yokD_2
ygaV
blaTEM-1
tnpR
neo_1
neo_2
smc
dcm
yhcR
ant1_1
xerD_1
hin_2
hin_1
IS26
blaNDM-1
qnrS1
bcr_2
umuD_1
blaOXA-10
aadA1
dhfrI
dfrA14
tetA
cynR
bcr_1
pinR
IS26
B
pS6-NDM-5
p3M-23
112 kbp
108 kbp
104 kbp
IS26
IS26
umuD_1
DNA polymerase V
hypothetical protein
IS3000
hypothetical protein
IS5
blaNDM-5
ble
IS26
C
GC Content
GC skew
GC skew(-)
GC skew(+)
pLJP3_1
100% identity
70% identity
50% identity
pTQ28-tet(X4)
100% identity
70% identity
50% identity
pS20-tet(X4)
186003 bp
20 kbp
40 kbp
60 kbp
80 kbp
100 kbp
120 kbp
140 kbp
160 kbp
180 kbp
traC
virB
parM
repB_2
lnu(G)
uvrD
dam
cobS
repB_1
tus
hha
higB-1
repE
rdmC
tet(X4)
floR
cynR
flmA
qnrS1
hin
tnpR
blaTEM-1
sppA
xerD
aadA22
hns
smc
resA
dcm
yhcR
dsbC
umuC
umuD
D
Tn2
pS76-2
repB
ygiW
fimD
faeE
rcsB
yjiK
gadX
finO
traA
traM
tnpA
tnpR
blaTEM-1
umuC
parA
rpnA
smc
cynT
mkaC
mkaB
spvB
spvC
xerD
ccdB
pS76-3
blaTEM-1
aph(6)-Id
aph(3'')-Ib
sul2
100%
30%
