# Supplementary material for: Genomic epidemiology and antimicrobial resistance of non-typhoidal Salmonella in children in Hainan Province, China
Source: Microbiol Spectr. 2026 Sep 23;14(11):e01502-26. doi: 10.1128/spectrum.01502-26 (PMC13729223; doi:10.1128/spectrum.01502-26)
Supplement: Supplementary file 3 [file spectrum.01502-26-s0002.pdf]

A

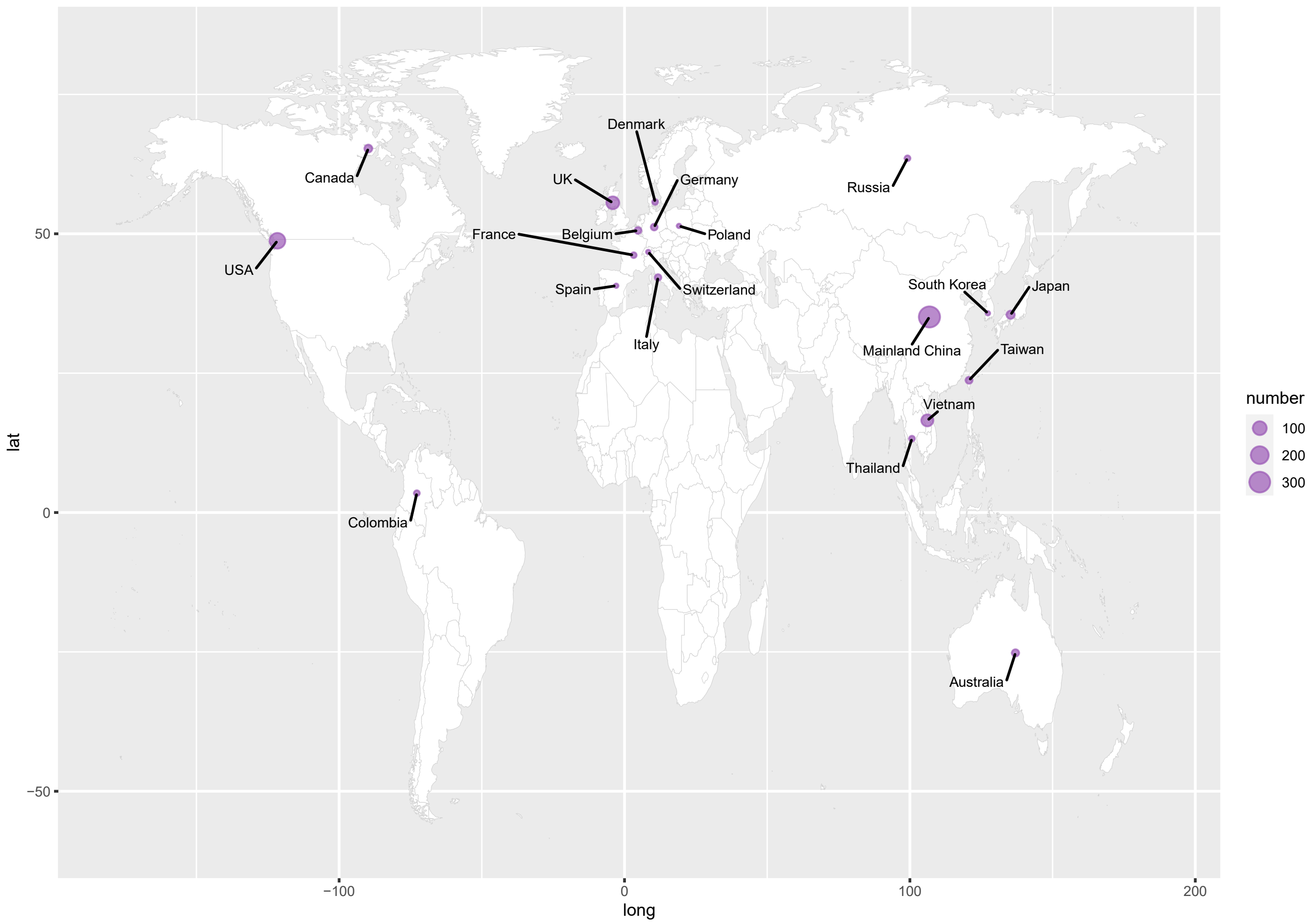
Denmark
Germany
UK
Canada
Russia
Poland
Belgium
France
USA
Switzerland
Spain
South Korea
Japan
Italy
Mainland China
Taiwan
Vietnam
Thailand
Colombia
Australia
number
100
200
300
lat
long
50
0
−50
−100
0
100
200

B

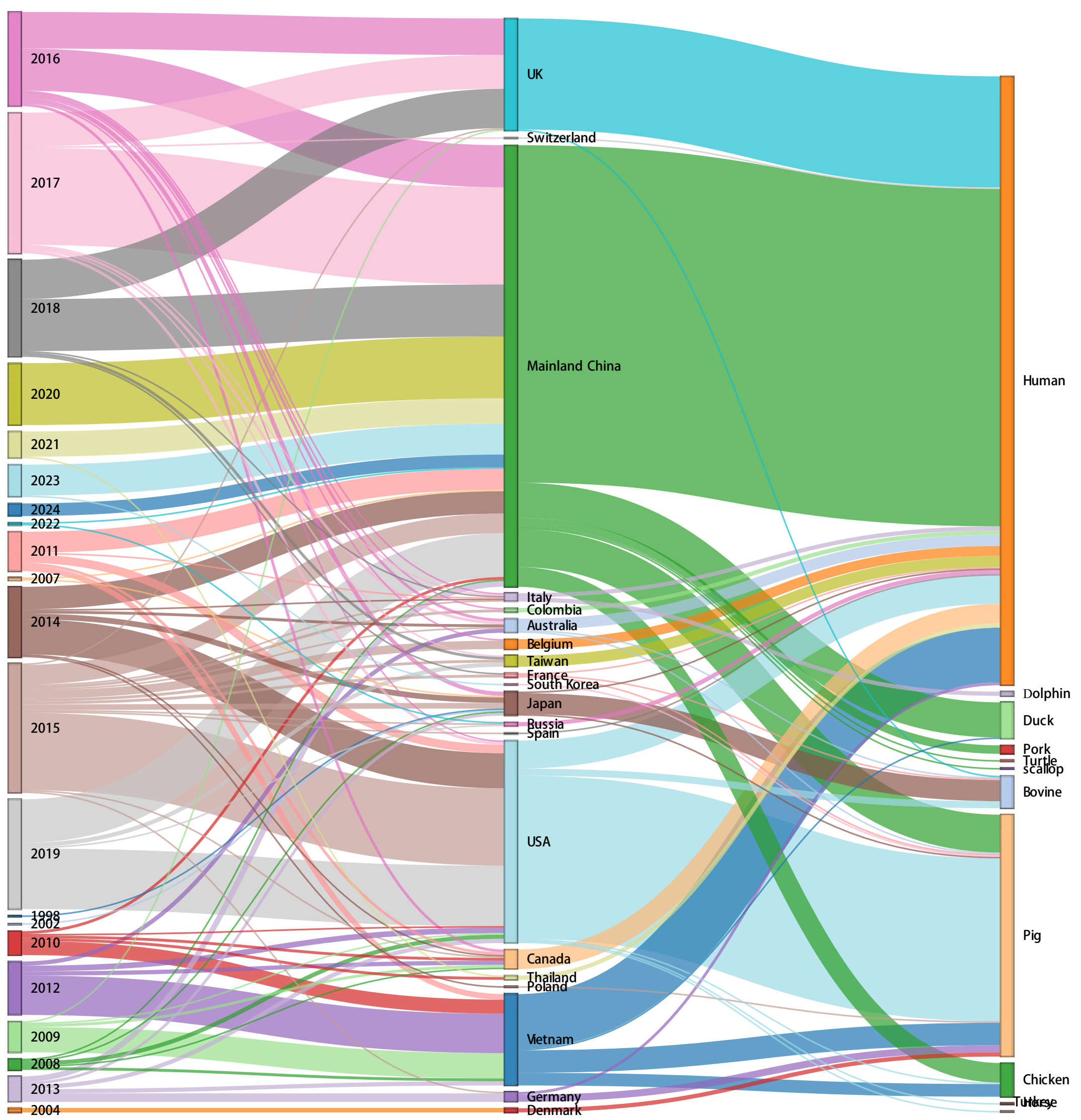
2016
2017
2018
2020
2021
2023
2024
2022
2011
2007
2014
2015
2019
1998
2002
2010
2012
2009
2008
2013
2004
UK
Switzerland
Mainland China
Italy
Colombia
Australia
Belgium
Taiwan
France
South Korea
Japan
Russia
Spain
USA
Canada
Thailand
Poland
Vietnam
Germany
Denmark
Human
Dolphin
Duck
Pork
Turtle
scallop
Bovine
Pig
Chicken
