# Supplementary material for: Genomic epidemiology and antimicrobial resistance of non-typhoidal Salmonella in children in Hainan Province, China
Source: Microbiol Spectr. 2026 Sep 23;14(11):e01502-26. doi: 10.1128/spectrum.01502-26 (PMC13729223; doi:10.1128/spectrum.01502-26)
Supplement: Supplementary file 4 [file spectrum.01502-26-s0003.pdf]

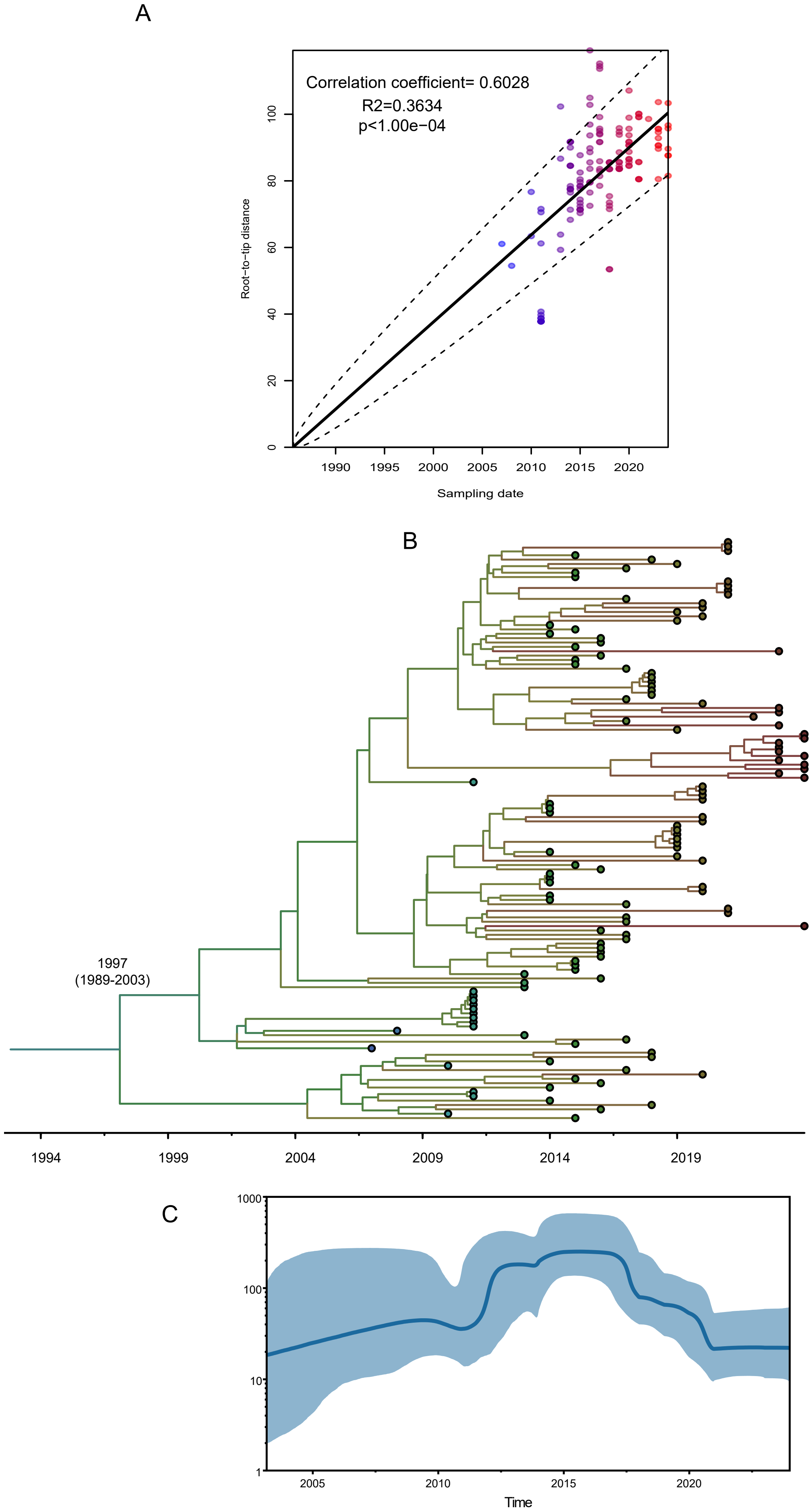
A
Correlation coefficient= 0.6028
R2=0.3634
p<1.00e−04
Root−to−tip distance
0
20
40
60
80
100
1990
1995
2000
2005
2010
2015
2020
Sampling date
B
1997
(1989-2003)
1994
1999
2004
2009
2014
2019
C
1000
100
10
1
2005
2010
2015
2020
Time
