# Supplementary material for: Genomic epidemiology and antimicrobial resistance of non-typhoidal Salmonella in children in Hainan Province, China
Source: Microbiol Spectr. 2026 Sep 23;14(11):e01502-26. doi: 10.1128/spectrum.01502-26 (PMC13729223; doi:10.1128/spectrum.01502-26)
Supplement: Supplementary file 5 [file spectrum.01502-26-s0004.docx]

**Table S1. Assembly quality metrics for the four hybrid-assembled strains**

| Isolate | S20 | S55 | S6 | S76 |
| --- | --- | --- | --- | --- |
| Contigs number | 2 | 5 | 2 | 3 |
| Largest contig (bp) | 4956067 | 5041665 | 5036698 | 4679746 |
| Total length (bp) | 5142070 | 5361625 | 5283867 | 4768557 |
| GC (%) | 51.99 | 51.86 | 51.91 | 52.13 |
| N50 (bp) | 4956067 | 5041665 | 5036698 | 4679746 |
| N90 (bp) | 4956067 | 5041665 | 5036698 | 4679746 |

**Table S3. Antimicrobial resistance rates (95% CI) by serotype in NTS.**

| Antimicrobials | I 4,[5],12:i:- (R%, 95% CI) | Typhimurium (R%, 95% CI) | *P** | Stanley (R%, 95% CI) | Derby (R%, 95% CI) | Weltevreden (R%, 95% CI) | Agona (R%, 95% CI) | Enteritidis (R%, 95% CI) | Goldcoast or Brikama (R%, 95% CI) | Oranienburg (R%, 95% CI) | Poona (R%, 95% CI) | Saintpaul (R%, 95% CI) | Other (R%, 95% CI) |
| --- | --- | --- | --- | --- | --- | --- | --- | --- | --- | --- | --- | --- | --- |
| AMP | 93.5, 78.6-99.2 | 83.3, 51.6-97.9 | 0.3080 | 33.3, 7.5-70.1 | 20.0, 0.5-71.6 | 0.0, 0.0-70.8 | 100.0, 15.8-100.0 | 100.0, 15.8-100.0 | 50.0, 1.3-98.7 | 0.0, 0.0-84.2 | 0.0, 0.0-84.2 | 100.0, 15.8-100.0 | 80.0, 28.4-99.5 |
| SAM | 77.4, 58.9-90.4 | 75.0, 42.8-94.5 | > 0.99 | 33.3, 7.5-70.1 | 20.0, 0.5-71.6 | 0.0, 0.0-70.8 | 100.0, 15.8-100.0 | 100.0, 15.8-100.0 | 0.0, 0.0-84.2 | 0.0, 0.0-84.2 | 0.0, 0.0-84.2 | 100.0, 15.8-100.0 | 80.0, 28.4-99.5 |
| CRO | 19.4, 7.5-37.5 | 33.3, 9.9-65.1 | 0.4267 | 33.3, 7.5-70.1 | 0.0, 0.0-52.2 | 0.0, 0.0-70.8 | 100.0, 15.8-100.0 | 0.0, 0.0-84.2 | 0.0, 0.0-84.2 | 0.0, 0.0-84.2 | 0.0, 0.0-84.2 | 100.0, 15.8-100.0 | 40.0, 5.3-85.3 |
| CAZ | 16.1, 5.5-33.7 | 33.3, 9.9-65.1 | 0.2371 | 33.3, 7.5-70.1 | 0.0, 0.0-52.2 | 0.0, 0.0-70.8 | 100.0, 15.8-100.0 | 0.0, 0.0-84.2 | 0.0, 0.0-84.2 | 0.0, 0.0-84.2 | 0.0, 0.0-84.2 | 0.0, 0.0-84.2 | 40.0, 5.3-85.3 |
| ETP | 9.7, 2.0-25.8 | 0.0, 0.0-26.5 | 0.5478 | 11.1, 0.3-48.2 | 0.0, 0.0-52.2 | 0.0, 0.0-70.8 | 0.0, 0.0-84.2 | 0.0, 0.0-84.2 | 0.0, 0.0-84.2 | 0.0, 0.0-84.2 | 0.0, 0.0-84.2 | 0.0, 0.0-84.2 | 0.0, 0.0-52.2 |
| IPM | 9.7, 2.0-25.8 | 0.0, 0.0-26.5 | 0.5478 | 0.0, 0.0-33.6 | 0.0, 0.0-52.2 | 0.0, 0.0-70.8 | 0.0, 0.0-84.2 | 0.0, 0.0-84.2 | 0.0, 0.0-84.2 | 0.0, 0.0-84.2 | 0.0, 0.0-84.2 | 0.0, 0.0-84.2 | 0.0, 0.0-52.2 |
| CIP | 16.1, 5.5-33.7 | 41.7, 15.2-72.3 | 0.1105 | 33.3, 7.5-70.1 | 0.0, 0.0-52.2 | 0.0, 0.0-70.8 | 0.0, 0.0-84.2 | 50.0, 1.3-98.7 | 0.0, 0.0-84.2 | 0.0, 0.0-84.2 | 0.0, 0.0-84.2 | 0.0, 0.0-84.2 | 20.0, 0.5-71.6 |
| LVX | 3.2, 0.1-16.7 | 0.0, 0.0-26.5 | > 0.99 | 0.0, 0.0-33.6 | 0.0, 0.0-52.2 | 0.0, 0.0-70.8 | 0.0, 0.0-84.2 | 0.0, 0.0-84.2 | 0.0, 0.0-84.2 | 0.0, 0.0-84.2 | 0.0, 0.0-84.2 | 0.0, 0.0-84.2 | 0.0, 0.0-52.2 |
| SXT | 61.3, 42.2-78.2 | 75.0, 42.8-94.5 | 0.4916 | 33.3, 7.5-70.1 | 20.0, 0.5-71.6 | 0.0, 0.0-70.8 | 0.0, 0.0-84.2 | 0.0, 0.0-84.2 | 50.0, 1.3-98.7 | 0.0, 0.0-84.2 | 0.0, 0.0-84.2 | 100.0, 15.8-100.0 | 60.0, 14.7-94.7 |
| TZP | 9.7, 2.0-25.8 | 0.0, 0.0-26.5 | 0.5478 | 33.3, 7.5-70.1 | 0.0, 0.0-52.2 | 0.0, 0.0-70.8 | 50.0, 1.3-98.7 | 0.0, 0.0-84.2 | 0.0, 0.0-84.2 | 0.0, 0.0-84.2 | 0.0, 0.0-84.2 | 0.0, 0.0-84.2 | 0.0, 0.0-52.2 |
| ATM | 9.7, 2.0-25.8 | 41.7, 15.2-72.3 | 0.0277 | 33.3, 7.5-70.1 | 0.0, 0.0-52.2 | 0.0, 0.0-70.8 | 100.0, 15.8-100.0 | 0.0, 0.0-84.2 | 0.0, 0.0-84.2 | 0.0, 0.0-84.2 | 0.0, 0.0-84.2 | 100.0, 15.8-100.0 | 20.0, 0.5-71.6 |
| MDR (%) | 74.2, 55.4-88.1 | 83.3, 51.6-97.9 | 0.6983 | 33.3, 7.5-70.1 | 20.0, 0.5-71.6 | 0.0, 0.0-70.8 | 100.0, 15.8-100.0 | 100.0, 15.8-100.0 | 50.0, 1.3-98.7 | 0.0, 0.0-84.2 | 0.0, 0.0-84.2 | 100.0, 15.8-100.0 | 80.0, 28.4-99.5 |

*Statistical comparison of resistance rates was limited to monophasic 1,4,[5],12:i:- and Typhimurium, the only serotypes with >10 isolates. Resistance proportions for remaining serotypes (n<10) are presented with exact 95% CI and should be interpreted with caution.

**Table S4. Sequence Type corresponding to each NTS serotype in 77 strains.**

| **Serogroup (n, %)** | **Serotype (n, %)** | **ST** |
| --- | --- | --- |
| B (61, 79.2) | I 4,[5],12:i:- (31, 40.3%) | ST34 |
|  | Typhimurium (12, 15.6%) | ST19 |
|  | Stanley (9, 11.7%) | ST29 |
|  | Derby (5, 6.5%) | ST40 |
|  | Agona (2, 2.6%) | ST13 |
|  | Saintpaul (2, 2.6%) | ST27 |
| C (7, 9.1) | Oranienburg (2, 2.6%) | ST23 |
|  | Goldcoast or Brikama (2, 2.6%) | ST358 |
|  | Rissen (1, 1.3%) | ST469 |
|  | Albany or Duesseldorf (1, 1.3%) | ST292 |
|  | Litchfield (1, 1.3%) | ST214 |
| D (3, 3.9) | Enteritidis (2, 2.6%) | ST11 |
|  | Javiana (1, 1.3%) | ST1547 |
| E (4, 5.2) | Weltevreden (3, 3.9%) | ST365 |
|  | Liverpool (1, 1.3%) | ST1959 |
| G (2, 2.6) | Poona (2, 2.6%) | ST2889 |

ST： Sequence Type

**Figure Legends**

**Figure S1. Genomic comparison of plasmids identified in this study.**

(A) Comparative analysis of the IncHI2A-type plasmids pS6-NDM-5 and pS55-NDM-5 with plasmids p3M-23 (China) and pZ1323SSL0063-1 (South Korea) retrieved from NCBI (B) Detailed view of the *bla*_NDM-5_-carrying region, which is present in pS6-NDM-5 but absent in p3M-23 (C) The IncFIA(HI1)/IncHI1A/IncHI1B(R27)-type plasmid pS20-tet(X4) harboring multiple resistance genes compared with plasmids from NCBI. (D) Scaled linear diagrams comparing the IncFIB(S)/IncFII(S)-type plasmid pS76-2 and the IncX1-type plasmid pS76-3. Grey shading and squares in colors indicate homologies between the corresponding genetic loci on each plasmid. Arrows indicate open reading frames, with arrowheads indicating the direction of transcription: red, antibiotic-resistant encoding genes. Other genes are shown as blue arrows.

**Figure S2. Global characteristics of the NTS ST34 dataset (n = 691).**

(A) Geographical distribution of 691 global ST34 isolates. (B) Sankey diagram depicting the interrelationships among isolation year, geographic region, and host source.

**Figure S3. Temporal and demographic analysis of the ST34 lineage.**

(A) Root-to-tip regression analysis. The correlation between root-to-tip genetic distance and sampling time (isolation year) indicates a relatively clock-like pattern of molecular evolution. The analysis was conducted on a subset of 139 genomes selected from the total dataset (n = 691) to optimize computational efficiency. (B) Time-calibrated maximum clade credibility (MCC) tree of the 139 global ST34 strains. (C) Bayesian skyline plot showing the effective population size dynamics of ST34 over time (logarithmic scale). The blue line indicates the median estimate, and the light blue shaded area represents the 95% highest posterior density (HPD) interval.
